# A Comparative Study of the Band-Edge Exciton Fine Structure in Zinc Blende and Wurtzite CdSe Nanocrystals

**DOI:** 10.3390/nano12234269

**Published:** 2022-12-01

**Authors:** Aleksandr A. Golovatenko, Ina V. Kalitukha, Grigorii S. Dimitriev, Victor F. Sapega, Maxim V. Rakhlin, Aidar I. Galimov, Tatiana V. Shubina, Elena V. Shornikova, Gang Qiang, Dmitri R. Yakovlev, Manfred Bayer, Amelie Biermann, Axel Hoffmann, Tangi Aubert, Zeger Hens, Anna V. Rodina

**Affiliations:** 1 Ioffe Institute, Russian Academy of Sciences, 194021 St. Petersburg, Russia; 2 Experimentelle Physik 2, Technische Universität Dortmund, 44221 Dortmund, Germany; 3 Institut für Festkörperphysik, Technische Universitat Berlin, 10623 Berlin, Germany; 4 Department of Chemistry, Ghent University, 9000 Ghent, Belgium

**Keywords:** exciton fine structure, colloidal nanocrystals, crystal structure, zinc blende, wurtzite, CdSe

## Abstract

In this paper, we studied the role of the crystal structure in spheroidal CdSe nanocrystals on the band-edge exciton fine structure. Ensembles of zinc blende and wurtzite CdSe nanocrystals are investigated experimentally by two optical techniques: fluorescence line narrowing (FLN) and time-resolved photoluminescence. We argue that the zero-phonon line evaluated by the FLN technique gives the ensemble-averaged energy splitting between the lowest bright and dark exciton states, while the activation energy from the temperature-dependent photoluminescence decay is smaller and corresponds to the energy of an acoustic phonon. The energy splittings between the bright and dark exciton states determined using the FLN technique are found to be the same for zinc blende and wurtzite CdSe nanocrystals. Within the effective mass approximation, we develop a theoretical model considering the following factors: (i) influence of the nanocrystal shape on the bright–dark exciton splitting and the oscillator strength of the bright exciton, and (ii) shape dispersion in the ensemble of the nanocrystals. We show that these two factors result in similar calculated zero-phonon lines in zinc blende and wurtzite CdSe nanocrystals. The account of the nanocrystals shape dispersion allows us to evaluate the linewidth of the zero-phonon line.

## 1. Introduction

Quantum confinement in zero-dimensional semiconductor nanocrystal quantum dots (NCs) was discovered 40 years ago [1,2,3]. Today, there are two main strategies in the fabrication of quantum dots: physical vacuum-based methods resulting in epitaxial quantum dots and chemical synthesis of colloidal NCs [4]. Comparison of the properties and applications of quantum dots made by these two methods can be found in Ref. [5]. The colloidal NCs remained the focus of extensive research last decade due to many related prospects in terms of nanoscience and nanotechnology [6,7]. Moreover, the variety of the material systems allowing the colloidal synthesis of NCs has been greatly extended to include—in addition to well-developed II-VI and III-V semiconductor NCs—the lead halide perovskite semiconductor NCs [8,9] as well as carbon-based nanocolloids [10]. Even for conventional semiconductor colloidal NCs, there are possibilities to combine different materials into core–shell heterostructures of various sizes and shapes [7], thus expanding the possibilities for new phenomena and applications. Recently, the family of CdSe colloidal NCs comprising nearly spherical nanocrystal quantum dots, nanorods, nanoplatelets, and tetrapods was replenished by cube-shaped nanocrystals [11]. Besides the shape, one can choose the desired crystal structure of the nanocrystals. While bulk CdSe has a wurtzite crystal structure, colloidal CdSe NCs can have either a wurtzite (wz) or zinc blende (zb) crystal structure depending on the synthesis conditions [12,13,14,15]. This additional freedom opens the possibility of studying the effect of the crystal structure on the optical properties of NCs. Of particular interest is the comparison of the fine structure of the band-edge exciton in zb- and wz-CdSe NCs and the possible verification of the theoretical predictions of their difference first made in Ref. [16].

**Figure 1:**
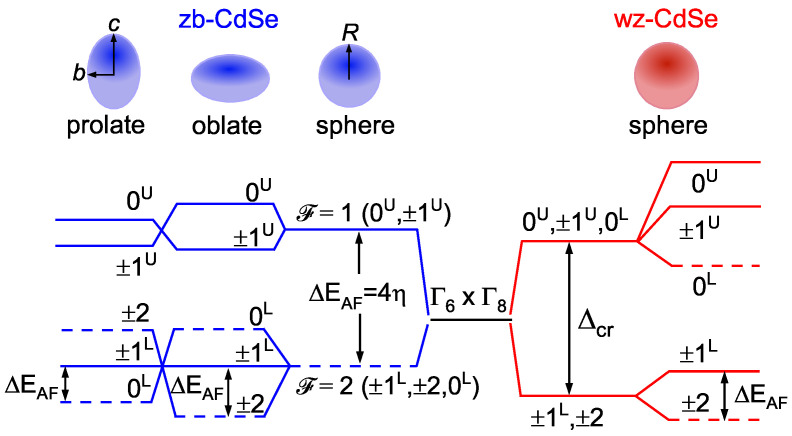
Schematic of the fine structure of the band-edge exciton formed by an electron from the $\Gamma_{6}$ conduction band and a hole from the $\Gamma_{8}$ valence band in spheroidal zb-CdSe NCs with different semiaxes aspect ratio c/b, including the spherical with NC radius R=c=b, and spherical wz-CdSe NCs. Solid and dashed lines correspond to bright and dark exciton states, respectively. wz-CdSe NCs are considered within the quasi-cubic approximation, i.e., as zb-CdSe NCs with split valence sub-bands of heavy and light holes.

The theoretical analysis of the fine structure of the band-edge exciton in CdSe NCs was performed within the effective mass approximation (EMA) [16,17,18], and using pseudopotential [19,20] and tight-binding methods [21]. All these methods predict similar fine structures for spherical wz-CdSe NCs: five eigenstates $\pm 2,\;\pm 1^{L},\;0^{L},\;\pm 1^{U},\;0^{U}$ (according to the notation from Ref. [16]) listed in ascending order of energy, as it is shown in Figure 1. In the electric-dipole approximation, states $\pm 2,\;0^{L}$ are optically inactive (dark excitons) while the states $\pm 1^{L},\;\pm 1^{U},\;0^{U}$ are optically active (bright excitons). Note that the dark excitons can still have a finite lifetime due to the admixture of bright states [22]. In the case of spherical zb-CdSe NCs, these theoretical methods predict a fine structure quite different from wz-CdSe NCs. Due to the absence of the crystal field splitting $\Delta_{\mathrm{cr}}$ between the heavy hole and light hole valence sub-bands, there are only two eigenstates that are characterized by the total angular momentum of electron and hole, F=1 (bright exciton) and F=2 (dark exciton). The splitting between these two states equals 4η with η being the effective electron–hole exchange interaction constant. However, the states F=1, 2 split into five states in spheroidal NCs of oblate or prolate shape, as shown in Figure 1. In oblate zb-CdSe NCs, the fine structure is similar to wz-CdSe NCs with ±2 state being the lowest state. In prolate zb-CdSe NCs, the lowest state is the $0^{L}$ state. Additional information about the fine structure can be found in the Supporting Information S1.

In wz-CdSe NCs, the fine structure of the band-edge exciton was studied in detail on the ensemble and single-NC level [16,23,24,25,26,27,28,29,30,31,32,33,34,35]. In these studies, the energy splitting between the lowest bright and dark exciton states $\Delta E_{\mathrm{AF}}$ was determined from: (i) the spectral shift between the zero-phonon line (ZPL) and the laser photon energy in fluorescence line narrowing (FLN) or photoluminescence excitation (PLE) spectroscopy [16,23,24,25,26,27,28]; (ii) the activation energy in the temperature dependence of the long-lasting component of photoluminescence (PL) decay as measured on the ensemble [27,28,29,30,31] and single NC level [32,33]; and (iii) the energy splitting between PL lines for single NCs [33,34,35].

In FLN/PLE studies, $\Delta E_{\mathrm{AF}}$ reaches 25 meV for an NC radius R=1.5 nm [24]. At the same time, $\Delta E_{\mathrm{AF}}$ determined from the temperature dependence of the PL decay within the frame of a model considering the thermal population of the bright and dark exciton states is in the few meV range [27,28,31,32]. The spectroscopy of single-core/shell CdSe/ZnS NCs allows the observation of the fine structure states directly, where the reported values of the dark–bright splitting agree with FLN/PLE data for a core radius R≥2.5 nm [34]. For single CdSe/ZnS NCs with a core radius R=1.5 nm, the evaluation of $\Delta E_{\mathrm{AF}}$ was done by the analysis of the temperature dependence of the PL decay [32]. The obtained $\Delta E_{\mathrm{AF}}=5$ meV is small compared to the 25 meV from the FLN/PLE data for bare-core CdSe NCs of the same radius. These results clearly reveal a contradiction between the $\Delta E_{\mathrm{AF}}$ determined from the spectral positions of exciton states and from the temperature dependence of the PL dynamics.

This contradiction was previously addressed in Refs. [28,29,36] in different ways. In Refs. [29,36], the $\Delta E_{\mathrm{AF}}$ from FLN/PLE studies was considered as the true splitting of the bright and dark excitons. The change in the PL decay lifetime at T<20 K was associated with a mechanism having a small activation energy of about 1 meV, close to the energy of the lowest quantized acoustic phonon mode. On the contrary, the $\Delta E_{\mathrm{AF}}$ from PL decay studies was considered as the bright–dark splitting in Ref. [28]. The large $\Delta E_{\mathrm{AF}}$ value from FLN/PLE was explained as a result of the exchange interaction of the exciton with polarized surface spins in Refs. [28,37].

While the majority of experimental data on the fine structure in wz-CdSe NCs was obtained by the FLN/PLE method, no such studies were performed for zb-CdSe NCs, for which $\Delta E_{\mathrm{AF}}$ so far was determined from the PL decay temperature dependence [31] and single NC spectroscopy [38,39]. The $\Delta E_{\mathrm{AF}}$ determined from the temperature dependence of the PL decay was found to be the same in zb- and wz-CdSe NCs of the same size [31]. This result was interpreted within the effective mass approximation as reflecting the similar splitting between the $0^{L}$ and $\pm 1^{L}$ states in prolate NCs of both types. Single NC studies were performed for core/shell/shell CdSe/CdS/ZnS NCs with a core radius R=2.7 nm and varying shape anisotropy [38,39]. The observed splittings between the two lowest emitting states in these NCs do not exceed 2 meV. Such a small splitting and the complex core/shell/shell structure do not allow one to draw a conclusion about the difference between the fine structure of zb- and wz-CdSe NCs. A comparison of small bare-core NCs with increased electron–hole exchange interaction is required for this task.

In this paper, we study the band-edge exciton fine structure in ensembles of zb- and wz-CdSe NCs. We compare the bright–dark splittings $\Delta E_{\mathrm{AF}}$ determined from the FLN/PLE and time-resolved PL methods. We argue that the zero-phonon line in FLN/PLE gives the ensemble-averaged splitting $\Delta E_{\mathrm{AF}}$, while the activation energy in the temperature-dependent PL decay is smaller and corresponds to the energy of an acoustic phonon. We show that the bright–dark splitting in zb- and wz-CdSe NCs show a similar dependence on the NC radius. To explain this result, we propose a theoretical model accounting for varying bright–dark splitting and oscillator strength of the bright exciton in an ensemble of NCs with shape dispersion. We show that these two factors result in similar calculated ZPL in zb- and wz-CdSe nanocrystals. We also show that an account of the NC shape dispersion allows for the evaluation of the ZPL linewidth.

## 2. Materials and Methods

### 2.1. Samples Synthesis

CdSe nanocrystals of wurtzite and zinc blende structure were synthesized according to the protocols described in Refs. [40,41], respectively. The radius of the NCs varies in the range of 1.5–2.5 nm. A comparative study of room temperature UV–Vis absorption spectra and X-ray diffractograms of zb- and wz-CdSe NCs under investigation can be found in Ref. [15].

#### 2.1.1. zb-CdSe NCs Synthesis

The CdSe NCs with predominantly zinc blende structure were synthesized according to a procedure based on the injection of undissolved Se powder into a hot mixture of cadmium carboxylate in octadecene (ODE) [41]. Briefly, CdO (0.5–1 mmol) is dissolved with a fatty acid (3 Cd equivalent) in ODE (10 mL) at 260 °C under air atmosphere. A solution of undissolved Se powder (0.1 Cd equivalent) in ODE (1 mL) is rapidly injected and the reaction is left to proceed for 5 min. The NCs are then purified by repeated centrifugation, using toluene and methanol as solvent and non-solvent, respectively. For this high chemical yield synthesis, the size of the NCs was varied by changing the length of the fatty acid from nonanoic acid to behenic acid, with longer acid chains yielding smaller nanocrystals, and/or the overall concentration of the synthesis, with higher concentrations yielding larger nanocrystals [41]. After synthesis, the surface ligands were systematically exchanged for oleic acid.

#### 2.1.2. wz-CdSe NCs Synthesis

The wurtzite CdSe QDs were synthesized according to a procedure reported by Carbone et al. [40]. In a typical synthesis, CdO (1 mmol) is dissolved with tetradecylphosphonic acid (2 mmol) in degassed trioctylphosphine oxide (6 g) at 350 °C under nitrogen. Solutions of trioctylphospine (TOP, 1 mL), followed by TOP-Se (1.7 M, 1 mL, Se fully dissolved beforehand at 60 °C in a glove box), are rapidly injected in the reaction mixture under nitrogen. The QDs size was adjusted by varying the reaction time from 2 s to 40 s. The QDs are finally purified by repeated centrifugation, using toluene and methanol as solvent and non-solvent, respectively.

### 2.2. Methods

For the FLN/PLE studies, a Hg high-pressure lamp combined with parallelized optics and a monochromator was used. With a slit width of 1 mm and a grating of 150 mm${}^{-1}$ in the monochromator, the excitation light spectrum can be narrowed down to 5 meV in the spectral range used in this work. The spectrally shaped light is focused on the sample, which is positioned in a helium flow cryostat. The signal emitted from the sample is collected under a small angle (about 10°) relative to the excitation direction (approximate back-scattering geometry) and subsequently spectrally decomposed in a monochromator (1800 mm${}^{-1}$ grating, 180 μm slit width) and detected with a CCD. For the measurement, the excitation energy is tuned stepwise and spectra are recorded with a fixed detection monochromator position. This results in effectively two-dimensional data plots, with the detected signal energy defining one parameter axis, stacked according to the excitation energy.

FLN spectra were also measured using resonant excitation by the different emission lines of a continuous wave Ar ion laser (465.8 nm, 476.5 nm, 488 nm, 514.5 nm). For this purpose, the samples were mounted in the variable temperature insert of a helium bath cryostat. The measurements were performed at T=1.6 K. The scattered light was detected in backscattering geometry, dispersed with a double monochromator equipped with a liquid nitrogen-cooled CCD camera.

The PL decay curves were measured at temperatures ranging from 4 K to 60 K using a ST-500-Attocube cryostat supplied with a temperature controller. For excitation, we used a picosecond-pulsed semiconductor laser PILAS 405 nm (Advanced Laser Systems) with an average excitation power of 100 nW measured before the cryostat window. A single-photon avalanche photodiode (SPAD) PDM 100 (Micro Photon Devices) with a time resolution of 40 ps was chosen as a detector, interfaced with the time-correlated single-photon counting system SPC-130 (Becker & Hickl).

The low-temperature absorption spectra of CdSe NCs were recorded with an Agilent Cary 6000i UV–Visible-NIR spectrophotometer combined with a helium flow cryostat.

## 3. Experimental Results

### 3.1. Bright–Dark Splitting Measured by FLN and PLE

**Figure 2:**
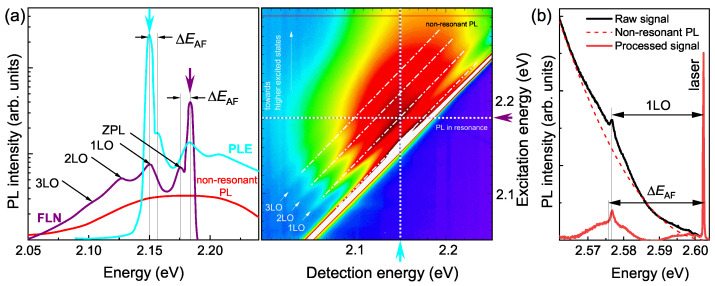
(**a**) FLN/PLE spectra obtained using excitation by a Hg lamp. (**Left panel**) FLN and PLE spectra measured with excitation and detection energies marked by the purple and cyan arrows in the hyperspectrum. (**Right panel**) FLN/PLE hyperspectrum for zb-CdSe NCs with a mean radius R=2 nm. (**b**) FLN spectrum for zb-CdSe NCs with R=1.6 nm obtained using excitation by an Ar ion laser. The shifts of the optical phonon-scattered laser light and dark exciton emissions are marked as 1LO and $\Delta E_{\mathrm{AF}}$, respectively.

First, the bright–dark splitting $\Delta E_{\mathrm{AF}}$ was obtained from the position of the ZPL in FLN and PLE spectra at T=5 K, using the Hg lamp combined with a monochromator for resonant excitation. The size dispersion of the NCs in an ensemble results in a broad non-resonant PL spectrum with a typical full-width at half-maximum (FWHM) of 70 meV (see the red trace in the left panel of Figure 2a). The resonant excitation in the FLN/PLE methods allows the observation of narrow lines from NCs of a certain size (see the cyan and purple traces in the left panel of Figure 2a). The combined FLN/PLE hyperspectrum for zb-CdSe NCs with R=2 nm is shown in the right panel of Figure 2a. The high-intensity white line corresponds to a scattered Hg lamp light. The four parallel dashed-dot lines in the hyperspectrum show the maxima of the dark exciton emission without the assistance of optical phonons (ZPL) and with the emission of 1–3 optical phonons (1LO–3LO). The optical phonon lines are superimposed on a broad background spectrum, which corresponds, according to Refs. [17,18,23], to the emission of the dark exciton after energy relaxation from the initially excited $0^{U}$ and $\pm 1^{U}$ states.

**Figure 3:**
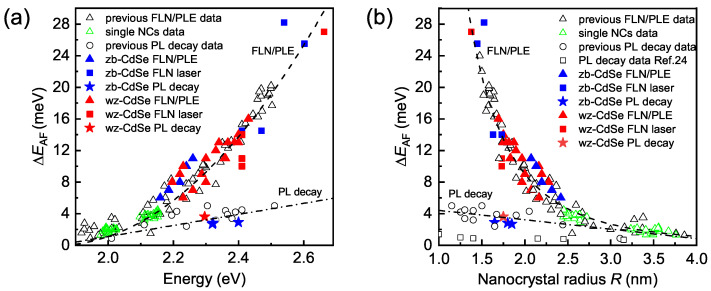
(**a**) Comparison of $\Delta E_{\mathrm{AF}}$ values determined by the FLN/PLE and PL decay methods (stars) for zb-CdSe (blue) and wz-CdSe (red) NCs. The FLN/PLE data obtained using excitation by the Hg lamp and the Ar ion laser are shown by triangles and squares, respectively. Open black and green triangles show the previous results of FLN/PLE [23,25,26,27] and single NCs studies [34], respectively. Open circles show previous results from PL decay studies [27,28,31,32]. For the FLN/PLE studies, the energy corresponds to the excitation/detection energy. For the PL decay studies, the energy corresponds to the maximum of the PL spectrum. The FLN/PLE data follow the trend $\Delta E_{\mathrm{AF}}\left(E\right)=\alpha_{E}{\left(E-E_{g}\right)}^{2}$ with the band gap energy $E_{g}=1841$ meV of wz-CdSe, and $\alpha_{E}=0.44\times 10^{-4}$ meV${}^{-1}$. (**b**) Dependence of the $\Delta E_{\mathrm{AF}}$ values presented in panel (**a**) on the NC radius *R*. The joint FLN/PLE data are complemented by data points from Ref. [24] which lack information about the excitation energy. For the $\Delta E_{\mathrm{AF}}$ determined from the PL decay studies, we use the nominal NC radii reported in Refs. [27,28,31,32]. Empty squares in panel (**b**) show the PL decay data from Ref. [30]. The FLN/PLE data follow the trend $\Delta E_{\mathrm{AF}}\left(R\right)=\alpha_{R}{\left(a_{B}/R\right)}^{3}$ with the exciton Bohr radius $a_{B}=5.6$ nm of CdSe, and $\alpha_{R}=0.44$ meV. Dash-dotted lines in panels (**a**,**b**) are guides for the eye for the PL decay data.

The dependencies of $\Delta E_{\mathrm{AF}}$ on the excitation/detection energy in the FLN/PLE spectra of zb-CdSe (blue triangles) and wz-CdSe (red triangles) NCs are shown in Figure 3a. As one can see, the $\Delta E_{\mathrm{AF}}$ values in both types of CdSe NCs show similar dependencies on excitation energy and coincide with the results of previous FLN/PLE studies [23,25,26,27] (empty black triangles) and single-NC studies [34] (empty green triangles). We note that $\Delta E_{\mathrm{AF}}$ is usually plotted as a function of NC radius. However, this approach depends on the used relationship between *R* and the excitation energy, which results in different dependencies $\Delta E_{\mathrm{AF}}\left(R\right)$ reported in the literature [23,25,26,27]; we avoid this problem using the optical energy as the abscissa. In Figure 3b, we plot $\Delta E_{\mathrm{AF}}\left(R\right)$ using the expression for the relationship between the energy of the low-temperature absorption maximum and the NC radius determined by the small-angle X-ray scattering (SAXS) [42]:

(1) $$\begin{array}{c} R\left(E\right)=R_{1}\frac{E_{0}}{2\sqrt{E(E-E_{0})}}, \end{array}$$

where $E_{0}=1.826$ eV is the low-temperature optical band gap (energy of the exciton resonance) of bulk CdSe and $R_{1}=2.25$ nm is received as the fitting parameter in Ref. [42]. The comparison of the experimental data and dependence R(E) given by Equation (2) is shown in Figure S1 of Supporting Information S2.

The obtained dependence $\Delta E_{\mathrm{AF}}\left(R\right)$ is in good agreement with all previous papers except Refs. [16,25]. As mentioned in Ref. [43], the NC radius determined using SAXS is on average 0.25 nm larger compared to the transmission electron microscopy value. We checked that the dependence $\Delta E_{\mathrm{AF}}\left(R\right)$ from Figure 3b coincides with the dependencies from Refs. [16,25] after the substitution R→R−0.25 nm (for details, see Supporting Information S2, Figure S2).

We extend the analysis to a set of small-sized (R= 1.4–1.8 nm) wz-CdSe and zb-CdSe NCs, resonantly excited by the Ar ion laser. In Figure 2b, one can see an FLN spectrum of zb-CdSe NCs with R=1.6 nm, excited at 2.602 eV photon energy. After the subtraction of the non-resonant PL background, we observe two peaks. The narrow peak corresponds to LO-phonon-scattered laser light. The broad peak (FWHM ≈10 meV) with the maximum at 2.576 eV corresponds to the emission of the dark exciton. The $\Delta E_{\mathrm{AF}}$ values obtained with laser excitation are shown by the blue and red squares in Figure 3a,b for the zb- and wz-CdSe NCs, respectively. For excitation energies below 2.5 eV, we find agreement with the FLN/PLE data obtained using excitation by the Hg lamp. For laser energies above 2.5 eV, the observed ZPL is centered near the LO-scattered laser light without pronounced dependence on the excitation energy. Again, we observe no significant difference between the zb- and wz-CdSe NCs.

### 3.2. Bright–Dark Splitting Evaluated from Temperature-Dependent PL Decay

**Figure 4:**
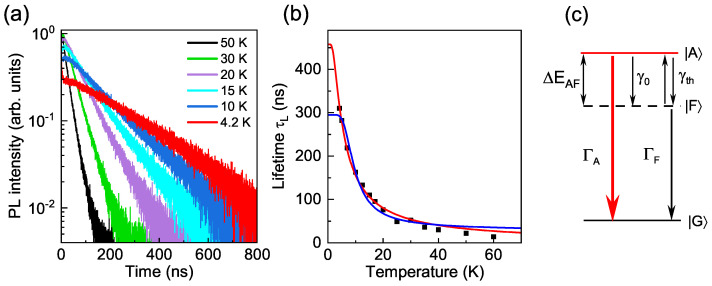
(**a**) PL decay measured at 4.2–50 K for zb-CdSe NCs with R=1.85 nm. (**b**) Temperature dependence of the long PL decay lifetime (black squares) for the NCs from panel (**a**). Blue line shows the fit by Equation (2) with fitting parameters $\tau_{F}=295$ ns, $\tau_{A}=14.2$ ns, $\Delta E_{\mathrm{AF}}=2.7$ meV. Red line shows the fit by Equation (3) based on the model considering the acoustic phonon activation of the dark exciton recombination [22] with parameters $\tau_{\mathrm{AC}}=458$ ns and $E_{\mathrm{AC}}=0.6$ meV. (**c**) Conventional three-level model for the determination of the splitting $\Delta E_{\mathrm{AF}}$ between bright |A〉 and dark |F〉 exciton states.

As mentioned above, the analysis of the temperature dependence of the PL decay is an alternative method for the determination of $\Delta E_{\mathrm{AF}}$. Here, we compare the temperature dependencies of the PL decay in zb- and wz-CdSe NCs with R= 1.6–1.8 nm. An example of a typical temperature dependence of the PL decay is shown in Figure 4a for zb-CdSe NCs with R=1.85 nm. We fit the temperature dependence of the long PL decay lifetime $\tau_{L}$ (see blue line in Figure 4b) within the conventional three-level scheme [32], as shown in Figure 4c:

(2) $$\begin{array}{c} \tau_{L}={\left[\frac{\Gamma_{A}+\Gamma_{F}}{2}-\frac{\Gamma_{A}-\Gamma_{F}}{2}\tanh\left(\frac{\Delta E_{\mathrm{AF}}}{2kT}\right)\right]}^{-1}, \end{array}$$

where $\Gamma_{A}=\tau_{A}^{-1}$ and $\Gamma_{F}=\tau_{F}^{-1}$ are the recombination rates of the bright |A〉 and dark |F〉 states to the ground state |G〉, respectively. $\gamma_{\mathrm{th}}$ is the thermalization rate due to the interaction with acoustic phonons. For the zero temperature relaxation rate $\gamma_{0}$ from |A〉 to |F〉, the relationship $\gamma_{0}\gg \Gamma_{A}$ must be fulfilled.

Using this approach, we obtain $\Delta E_{\mathrm{AF}}\approx 3$ meV for both types of CdSe NCs, in good agreement with Ref. [31]. The current and previous $\Delta E_{\mathrm{AF}}$ values obtained from the temperature dependence of the PL decay are shown by stars and open circles in Figure 3a, respectively. The dependence of $\Delta E_{\mathrm{AF}}$, determined from PL decay studies, on the NC radius is shown in Figure 3b. Here, data points from Ref. [30] (empty squares) are additionally added, compared to Figure 3a. Again, we do not observe a significant difference between zb-CdSe and wz-CdSe NCs, both in the FLN/PLE and in the PL decay studies. However, the difference between the $\Delta E_{\mathrm{AF}}$ values determined within these two methods strongly increases for exciton energies exceeding 2.2 eV, corresponding to NC radii R<2.5 nm.

## 4. Theoretical Modeling

### 4.1. Alternative Modeling of the Temperature-Dependent PL Decay

According to Ref. [28], the difference between the $\Delta E_{\mathrm{AF}}$ values from the FLN/PLE and PL decay methods could be caused by the exchange interaction of the exciton with surface spins in small NCs. This interaction potentially gives an additional contribution to $\Delta E_{\mathrm{AF}}$ observable in FLN/PLE. If that were the case, CdSe NCs with different surface properties should have different $\Delta E_{\mathrm{AF}}$ values. As one can see in Figure 3, the FLN/PLE studies reveal the same $\Delta E_{\mathrm{AF}}$ within the whole range of excitation energies (NC sizes) in zb- and wz-CdSe NCs, CdSe NCs with different ligands [25], and CdSe NCs in a glass matrix [24,26,27]. Furthermore, as shown in Ref. [36], the $\Delta E_{\mathrm{AF}}$ determined from the FLN/PLE method exceeds the $\Delta E_{\mathrm{AF}}$ from PL decay studies in CdTe and InAs NCs. Since a universal effect of polarized surface spins in all these cases is doubtful, we consider another explanation of the difference between the two methods as proposed in Ref. [36].

In addition to the thermal population of the bright exciton state, as described by Equation (2), one should take into account the mechanism controlled by the fast and energetically favorable interaction with the phonon. In Ref. [36], it was shown that the $\Delta E_{\mathrm{AF}}$ determined from PL decay studies within the three-level model is close to the energy of the lowest acoustic phonon mode with an angular momentum of l=2. The activation of the dark exciton recombination by interaction with this phonon was considered in Ref. [22]. According to Ref. [22], the shortening of the PL decay lifetime is caused not by the thermal population of the bright exciton state or a vibronic state of the dark exciton [36], but by the acoustic phonon-induced admixture of the bright state. The temperature dependence of the ±2 state lifetime in this case is described by [22]:

(3) $$\begin{array}{c} \tau_{2}=\tau_{\mathrm{AC}}\tanh\left(E_{\mathrm{AC}}/2k_{B}T\right), \end{array}$$

where $\tau_{\mathrm{AC}}$ is the lifetime of the ±2 state at T=0 K provided by the emission of the acoustic phonon only, and $E_{\mathrm{AC}}$ is the energy of the acoustic phonon mode with l=2. A fit of the temperature dependence of the long PL decay component assuming $\tau_{L}=\tau_{2}$ is shown by the red line in Figure 4b. This model provides a better fit of the data with the fit parameter $E_{\mathrm{AC}}$ close to the energy of the l=2 acoustic phonon mode (see Supporting Information S5, Figure S12). A comparison of the three-level model and the model of acoustic phonon-induced mixing for several samples of CdSe NCs is given in Supporting Information S5, Figures S6–S11. Thus, following Refs. [29,36] we consider that $\Delta E_{\mathrm{AF}}$ from FLN/PLE studies reflects the true bright–dark exciton splitting, however, as we discuss in the next subsection, averaged over the ensemble of NCs with different shapes.

### 4.2. Theory of the FLN Spectra with Account for the Nanocrystal Shape Dispersion

Let us turn to the remaining puzzle: the same $\Delta E_{\mathrm{AF}}$ in zb- and wz-CdSe NCs. In a recent paper [20], the authors demonstrated with a pseudopotential method that the calculated splitting between F=1 and F=2 states in spherical zb-CdSe NCs should be close to the experimentally measured $\Delta E_{\mathrm{AF}}\left(R\right)$ dependence shown in Figure 3b. Thus, one could assume that the same $\Delta E_{\mathrm{AF}}$ in zb- and wz-CdSe NCs corresponds to the splitting between the ±2 and $\pm 1^{L}$ states in the wz-CdSe NCs and between the F=1 and F=2 states in the zb-CdSe NCs. However, this scenario has to be excluded because the non-resonant Stokes shift (energy difference between the first absorption and PL maxima) in both types of studied CdSe NCs is similar (see Supporting Information S3, Figures S3 and S4). The same result was found previously for the room temperature non-resonant Stokes shift in zb- and wz-CdSe NCs in Refs. [12,13]. The significant difference between the $\Delta E_{\mathrm{AF}}$ and the non-resonant Stokes shift indicates the presence of several bright exciton states with different energies in zb-CdSe NCs similar to the case in wz-CdSe NCs [16,23,24,25].

As the crystal field is absent in zb-CdSe, a possible interpretation of the similar fine structure in zb-CdSe and wz-CdSe involves a shape anisotropy of the zb-CdSe NCs (see Figure 1). Our estimations within the EMA show that zb-CdSe NCs of varying sizes have an oblate shape with an NC semiaxes ratio c/b≈0.9 to fit the $\Delta E_{\mathrm{AF}}\left(R\right)$ dependence from Figure 3b. However, it is known that if there is a shape anisotropy in zb- and wz-CdSe NCs, then it is predominantly an anisotropy of the prolate type [16,31]. For this reason, the introduction of an oblate NC shape as a fit parameter in the FLN/PLE data is not convincing. Below, we show that the similar ZPL signal in zb- and wz-CdSe NCs can be modeled if one considers a dependence of the $\pm 1^{L}$ bright exciton oscillator strength on the NC shape and a shape dispersion in an ensemble of nominally spherical zb-CdSe NCs.

Let us consider an ensemble of zb-CdSe NCs with a normal distribution of the NC shape anisotropy:

(4) $$\begin{array}{c} g\left(\mu \right)=\frac{1}{\sigma \sqrt{2\pi }}\exp\left[-\frac{{\left(\mu -\mu_{0}\right)}^{2}}{2\sigma^{2}}\right], \end{array}$$

where μ is the anisotropy parameter μ=c/b−1, $\mu_{0}$ is the mean anisotropy parameter for NCs of a given radius, and σ is the standard deviation.

**Figure 5:**
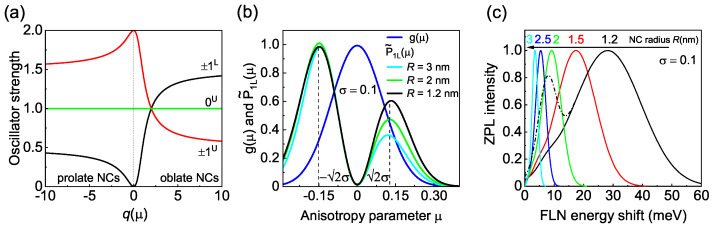
(**a**) Relative oscillator strengths of the $\pm 1^{L}$, $\pm 1^{U}$ and $0^{U}$ excitons depending on the parameter q(μ). (**b**) Anisotropy distribution g(μ) and weighted oscillator strength ${\tilde{P}}_{1^{L}}$ in zb-CdSe NCs with different radii for σ=0.1. (**c**) Shape of the zero phonon line in NCs of different sizes. The black dash-dotted line is calculated assuming equal emission efficiencies of the ±2 and $0^{L}$ states. Solid lines are calculated assuming that the emission efficiency from the $0^{L}$ is an order of magnitude smaller than that from the ±2 state.

The ZPL in FLN studies of CdSe NCs is the result of the dark exciton emission after resonant excitation of the $\pm 1^{L}$ bright exciton. We write the oscillator strengths of the $\pm 1^{L,U}$ states, measured in units of the oscillator strength of the $0^{U}$ state using Equation (28) from Ref. [16], in a slightly modified manner: [16]

(5) $$\begin{array}{c} P_{1^{L,U}}\left(\mu \right)=1\pm \frac{q(\mu )-2}{2\sqrt{q^{2}\left(\mu \right)-q\left(\mu \right)+1}}, \end{array}$$

where + and − correspond to the $\pm 1^{L}$ and $\pm 1^{U}$ states, respectively. q(μ)=Δ(μ)/4η, $\Delta \left(\mu \right)=\Delta_{\mathrm{cr}}+\Delta_{\mathrm{sh}}\left(\mu \right)$ is the joint splitting of the hole states with a projection of the total angular momentum on the anisotropy axis ±3/2 and ±1/2 due to the crystal field and NC shape anisotropy $\Delta_{\mathrm{sh}}\left(\mu \right)$ [16]. In the case of zb-CdSe NCs, $\Delta_{\mathrm{cr}}=0$ meV. The splitting 4η between the F=1,2 states is caused by the electron–hole short-range and long-range exchange interaction [17,18] with η=0.37 meV $\cdot {\left(a_{B}/R\right)}^{3}$, where $a_{B}=5.6$ nm is the exciton Bohr radius in CdSe. The dependence of the oscillator strengths on q(μ) is shown in Figure 5a for the $\pm 1^{U,L},0^{U}$ states. One can see that the finite oscillator strength of the $\pm 1^{L}$ exciton (black line in Figure 5a) in zb-CdSe is gained when the NC shape differs from a sphere.

The relative contribution to the ZPL signal ${\tilde{P}}_{1^{L}}\left(\mu \right)$ from NCs having an arbitrary μ is proportional to the fraction of these NCs, i.e., g(μ), and the oscillator strength of the $\pm 1^{L}$ state: ${\tilde{P}}_{1^{L}}\left(\mu \right)=P_{1^{L}}\left(\mu \right)g\left(\mu \right)$. The maximum of the ${\tilde{P}}_{1^{L}}\left(\mu \right)$ distribution determines a sub-ensemble of NCs which give the predominant contribution to the absorption of the light by the $\pm 1^{L}$ exciton.

Let us analyze zb-CdSe NCs with the maximum of the shape distribution corresponding to a sphere, i.e., $\mu_{0}=0$. In line with Ref. [44], which reported a standard deviation σ=0.18 for an ensemble of CdSe NCs with R=3 nm, for a first evaluation, we set σ=0.1. As can be seen in Figure 5b, the calculated ${\tilde{P}}_{1^{L}}\left(\mu \right)$ distribution has two maxima at $\pm \sqrt{2}\sigma$ corresponding to oblate (−) and prolate (+) NCs.

To calculate the ZPL spectrum, we plot ${\tilde{P}}_{1^{L}}$ as a function of $\Delta E_{\mathrm{AF}}$ using the relationship between $\Delta E_{\mathrm{AF}}$ and μ (see Supporting Information S1). According to this approach, one should observe two ZPLs corresponding to the splitting between the ±2 and $\pm 1^{L}$ states in oblate NCs, and to the splitting between the $0^{L}$ and $\pm 1^{L}$ states in prolate NCs. The results of a similar calculation of the ZPL spectrum in zb-CdSe NCs of different radii are shown in Figure 5c, again taking σ=0.1. If we consider equal efficiencies of emission from the $0^{L}$ and ±2 dark exciton states, the ZPL spectrum has two peaks (see the black dash-dotted line in Figure 5c). However, on single prolate zb-CdSe and wz-CdSe NCs, emission from the lowest $0^{L}$ state was only observed when a magnetic field was applied [35,39]. Probably, the activation of the $0^{L}$ state recombination in a zero magnetic field is weak, resulting in a correspondingly weak contribution to the ZPL. The results of ZPL calculations using the assumption that the emission from the ±2 state is an order of magnitude more efficient than the radiation from the $0^{L}$ state are shown by the solid lines in Figure 5c.

**Figure 6:**
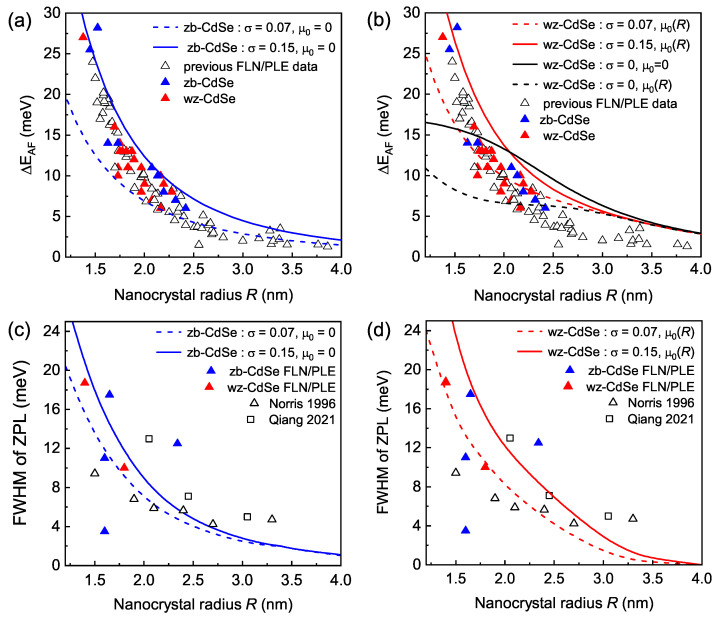
(**a**) Calculated energy of the ZPL maximum in an ensemble of zb-CdSe NCs for σ varying from 0.07 to 0.15. Symbols show the experimental FLN/PLE data from Figure 3b. Here, colored triangles show the joint set of data obtained using excitation by the Hg lamp and the Ar ion laser. (**b**) Calculated energy of the ZPL maximum in an ensemble of wz-CdSe NCs with σ varying from 0.07 (red dashed line) to 0.15 (red solid line) and with the size-dependent $\mu_{0}\left(R\right)$ from Ref. [16]. Solid and dashed black lines show calculated $\Delta E_{\mathrm{AF}}$ with σ=0 and $\mu_{0}=0$ or $\mu_{0}\left(R\right)$ from Ref. [16], respectively. (**c**) Calculated dependence of the ZPL linewidth on the NC radius for *σ* varying from 0.07 (dashed lines) to 0.15 (solid lines) in zb-CdSe NCs with $\mu_{0}=0$. Symbols show experimental values from the current and previous studies [23,27]. (**d**) Calculated dependence of the ZPL linewidth on the NC radius for *σ* varying from 0.07 (dashed lines) to 0.15 (solid lines) in wz-CdSe NCs with *μ*_0_(*R*) taken from Ref. [16].

From the positions of the maxima in the calculated ZPLs, we determined the $\Delta E_{\mathrm{AF}}$ splitting. In Figure 6a, we show the expected $\Delta E_{\mathrm{AF}}$ values in ensembles of zb-CdSe NCs with σ=0.07 and σ=0.15 giving the lower and the upper boundary, respectively. Clearly, the experimental data are placed well within these two theoretical limits. Using the same approach, we calculated the $\Delta E_{\mathrm{AF}}$ in an ensemble of wz-CdSe NCs with $\Delta_{\mathrm{cr}}=23$ meV, a similar shape dispersion and size-dependent $\mu_{0}\left(R\right)$ taken from Ref. [16]. The results of these calculations are shown by the red traces in Figure 6b. For comparison, we also show in Figure 6b the variation of $\Delta E_{\mathrm{AF}}$ calculated with σ=0 for spherical ($\mu_{0}=0$) wz-CdSe NCs [17,18] (the black solid line) and wz-CdSe NCs with $\mu_{0}\left(R\right)$ taken from Ref. [16] (the black dashed line). One can see that including the shape dispersion results in better agreement with the experimental data for small NCs with R<2.5 nm. However, for large NCs, the calculated $\Delta E_{\mathrm{AF}}$ still exceeds the experimental values.

Besides the position of the ZPL maximum, the dispersion of the NC shape in the ensemble allows for an evaluation of the ZPL linewidth. The comparison of the evaluated and measured linewidths is shown in Figure 6c,d. According to our model, in small NCs, the ZPL linewidth increases due to the larger effect of the shape anisotropy on the splitting of the hole states, $\Delta_{\mathrm{sh}}\left(\mu \right)\propto \mu R^{-2}$. As one can see, our estimations are in good agreement with the data from the current study and from Refs. [23,27].

## 5. Discussion

We have shown above that in the case of nearly spherical CdSe NCs, the FLN/PLE spectra exhibit the bright–dark $\Delta E_{\mathrm{AF}}$ splitting, and the temperature dependence of the PL decay gives an energy close to the energy of the acoustic phonon mode with l=2. Noticeably, in the case of colloidal CdSe nanoplatelets, both methods give similar $\Delta E_{\mathrm{AF}}$ values [45]. We see two reasons for this: (i) the dark exciton recombination is efficient through the admixture of the $0^{U}$ state by acoustic phonons in CdSe NCs; (ii) the large bright–dark splitting in small CdSe NCs with R<2.5 nm allows for acoustic phonon activation at T<20 K, while the thermal population of the bright exciton state requires T>20 K. Indeed, in NCs with R>2.5 nm, the difference between the $\Delta E_{\mathrm{AF}}$ determined from the FLN/PLE and PL decay methods vanishes, and it is difficult to distinguish between the thermal population of the bright exciton state and the acoustic phonon activation of the dark exciton state.

The comparison of the $\Delta E_{\mathrm{AF}}$ values from FLN/PLE studies revealed no significant difference between zb- and wz-CdSe NCs. We proposed an explanation of this observation based on the NC shape dispersion. This explanation assumes an ideal zinc blende or wurtzite crystal structure of the NCs. Previous comparative spectroscopic studies of zb- and wz-CdSe NCs [12,13,14,15] showed that both types of NCs of fixed size show almost the same energy of the first maximum in UV–Vis absorption spectra and only vary in the energy difference between the first and the second absorption peak maxima. Our study shows that the exciton fine structures of both types of CdSe NCs are also similar. While the proposed theoretical model demonstrates that, for certain conditions, zb- and wz-CdSe NCs can have similar FLN/PLE spectra, the question remains, namely whether NCs can be legitimately described as small pieces of a bulk semiconductor with a well-defined crystal structure. It was previously shown [14,46] that nominal wz-CdSe NCs can have several stacking faults, i.e., inclusions of zb-CdSe, even when the X-ray powder diffractogram still exhibits all the diffraction peaks of wz-CdSe [46]. Similarly, nominal zb-CdSe NCs can have inclusions of wz-CdSe. Such a mixed crystal structure, instead of pure zinc blende or pure wurtzite, could explain in a natural way the coincidence of the FLN/PLE data for both types of CdSe NCs. The advanced pseudopotential or tight-binding calculations of the exciton fine structure in CdSe NCs having stacking faults are required for the clarification of this interpretation.

Finally, to reveal the effect of crystal structure, we compared the $\Delta E_{\mathrm{AF}}$ determined from FLN/PLE studies for both types of CdSe NCs and for CdTe NCs [47,48], which are known to have a zinc blende crystal structure. The dependence of $\Delta E_{\mathrm{AF}}$ on the excitation energy for CdTe NCs behaves like the dependence for CdSe NCs shifted by the difference in the band gaps of CdSe ($E_{g}=1.841$ eV) and CdTe ($E_{g}=1.6$ eV) (see Supporting Information S4, Figure S5). This is clearly seen when we compare the dependencies of $\Delta E_{\mathrm{AF}}$ on the effective quantization energy equal to the difference between the excitation energy and the band gap. We also found similar dependencies of $\Delta E_{\mathrm{AF}}$ on the NC radius in the comparison of the data from Figure 3b with the data from Refs. [47,48]. In Refs. [47,48], the authors explained the $\Delta E_{\mathrm{AF}}$ as the splitting between the F=1 and F=2 exciton states. However, the zb-CdSe, wz-CdSe and zb-CdTe NCs have not only similar $\Delta E_{\mathrm{AF}}$ but also similar size-dependent non-resonant Stokes shifts [48] (see Supporting Information S3, Figure S4), which can be explained only for a fine structure that has several strongly split bright exciton states. As for zb-CdSe NCs, this requires an NC shape anisotropy or a built-in crystal field due to the inclusion of the wurtzite phase.

## 6. Conclusions

In conclusion, we have shown experimentally that the zb- and wz-CdSe NCs of the same size have identical fine structures of the band-edge exciton that can be determined by FLN/PLE spectroscopy. We developed a theoretical approach in order to analyze the role of the NC shape dispersion in an ensemble on the measured FLN/PLE spectra. The effect of shape dispersion allows us to calculate the experimental shift and linewidth of the zero phonon line in the zb- and wz-CdSe NCs. Despite the good agreement between our theoretical model and the experimental $\Delta E_{\mathrm{AF}}$ data, the question about the difference in the band-edge exciton fine structure in both types of CdSe NCs in the presence of stacking faults or other types of crystal structure distortions remains open.

## Data Availability

Not applicable.

## Supplementary Materials

The following supporting information can be downloaded at: https://www.mdpi.com/article/10.3390/nano12234269/s1. References [15,16,17,18,22,23,25,27,31,32,42,43,47,48] are cited in the supplementary materials. Figure S1: Fit of the low-temperature absorption; Figure S2: (a) Calculated energy of the ZPL maximum in an ensemble of zb-CdSe NCs for varying from 0.07 to 0.15. (b) Calculated energy of the ZPL maximum in an ensemble of wz-CdSe NCs with σ varying from 0.07 (red dashed line) to 0.15 (red solid line) and size dependent µ0(R); Figure S3: Low-temperature (T = 5 K) photoluminescence (black lines) and absorption spectra (red lines) in zb- and wz-CdSe NCs; Figure S4: Size dependence of the low-temperature non-resonant Stokes shifts in wz-CdSe NCs; Figure S5: Dependence of the bright-dark splitting Δ$E_{\mathrm{AF}}$ om (a) the excitation energy Eexc and (b) the effective quantization energy $E_{exc}-E_{g}$ for CdSe NCs with $E_{g}$ = 1.841 eV (empty triangles) and CdTe NCs with $E_{g}$ = 1.602 eV (c) Dependence of the Δ$E_{\mathrm{AF}}$ on the radius of CdSe NCs and CdTe NCs; Figure S6: Experimental temperature dependence of the long PL decay time $\tau_{L}$ (symbols) in zb-CdSe and wz-CdSe NCs; Figure S7: Experimental temperature dependence of the long PL decay rate τ${}^{-1}$ (symbols) in zb-CdSe and wz-CdSe NCs; Figure S8: Fits to the τ and τ${}^{-1}$ data; Figure S9: Fits to the $\tau_{L}$ data in wz-CdSe NCs with R = 1.65–3.05 nm; Figure S10: Fit to the τ${}^{-1}$ data in wz-CdSe NCs with R = 1.65–3.05 nm; Figure S11: Fits to the $\tau_{L}$ and τ${}^{-1}$ data measured on single CdSe NCs; Figure S12: Dependence of the energy EAC determined from fitting the $\tau_{L}$ (T) dependence within the model of acoustic phonon-induced activation. Supporting Information S1: The band-edge exciton ne structure; Supporting Information S2: Dependence of the Δ$E_{\mathrm{AF}}$ on the NC radius.
