# Supplementary material for: A Comparative Study of the Band-Edge Exciton Fine Structure in Zinc Blende and Wurtzite CdSe Nanocrystals
Source: Nanomaterials (Basel). 2022 Dec 1;12(23):4269. doi: 10.3390/nano12234269 (PMC9736692; doi:10.3390/nano12234269)
Supplement: Supplementary file 1 [file nanomaterials-12-04269-s001.zip › Supporting Information.pdf]

A comparative study of the band-edge exciton fine energy structure in zinc blende and wurtzite CdSe nanocrystals

A. A. Golovatenko[1], I. V. Kalitukha[1], G. S. Dimitriev[1], V. F. Sapega[1], M. V. Rakhlin[1], A. I. Galimov[1], T. V. Shubina[1], E. V. Shornikova[2], G. Qiang[2], D. R. Yakovlev[1,2], M. Bayer [1,2], A. Biermann[3], A. Hoffmann[3], T. Aubert[4], Z. Hens[4], and A. V. Rodina[1]

[1] *Ioffe Institute, St. Petersburg 194021, Russia*
[2] *Experimentelle Physik 2, Technische Universität Dortmund, 44221 Dortmund, Germany.*
[3] *Institut für Festkörperphysik, Technische Universitat Berlin, Berlin, Germany.*
[4] *Department of Chemistry, Ghent University, 9000 Ghent, Belgium.*

E-mail:

sasha.pti@mail.ioffe.ru

Supporting Information

S1.The band-edge exciton fine structure

According to Refs. [1,2], the relative energies of $\pm2, \pm0^{L,U}, \pm1^{L,U}$ exciton states are given by [1]:

$$\varepsilon_{\pm2} = -3\eta/2 - \Delta(\mu), \tag{S1}$$

$$\varepsilon_{0^{U,L}} = \eta/2 + \Delta(\mu)/2 \pm 2\eta, \tag{S2}$$

$$\varepsilon_{\pm1^{U,L}} = \eta/2 \pm \sqrt{(2\eta - \Delta(\mu))^2/4 + 3\eta^2}. \tag{S3}$$

where U and L correspond to $+$ and $-$, respectively. The parameters $\Delta(\mu)$ and η depend on the ratio of the light and heavy hole masses $\beta = m_{lh}/m_{hh}$. We use for both types of CdSe NCs $\beta = 0.28$ [1], corresponding to $m_{hh} = 1.13m_0$, where m_0 is the mass of the free electron. For the chosen β, the exchange interaction parameter $\eta = 0.37(a_B/R)^3$ meV, where $a_B = 5.6$ nm is the exciton Bohr radius in CdSe, and R is the NC radius. The parameter $\Delta(\mu) = \Delta_{\text{cr}} + \Delta_{\text{sh}}$ describes the splitting of the hole states with the projections of angular momentum $\pm3/2$ and $\pm1/2$ on the anisotropy axis. $\Delta_{\text{cr}} = v(\beta)\Delta_{\text{bulk}}$ is the contribution related to the crystal field splitting in wurtzite CdSe, which equals to $\Delta_{\text{bulk}} = 25$ meV in bulk CdSe. Using the dependence $v(\beta)$ [1] for a chosen β one finds $\Delta_{\text{cr}} = 23$ meV. Further, Δ_{sh} is the contribution arising from the splitting caused by the NC shape anisotropy. According to Ref. [1] it equals:

$$\Delta_{\text{sh}} = 2\mu u(\beta)\frac{\hbar^2\varphi^2(\beta)}{2m_{hh}R^2} \tag{S4}$$

where $\mu = c/b - 1$ is the anisotropy parameter, with c and b being the NC semiaxes. μ is positive (negative) in prolate (oblate) NCs. The function $u(\beta)$ describes the effect of the NC shape anisotropy that depends on β. For the chosen β value, $u(\beta) = -0.26$. $\varphi(\beta)$ is the root of the equation for radial wavefunctions [1]. For the chosen β, we find $\varphi(\beta) = 4.7$.

For $\Delta(\mu) > 0$, the lowest dark exciton state is the ±2 state. This is the case of wz-CdSe NCs or oblate zb-CdSe NCs. The splitting ΔE_{AF} between the lowest bright $\pm1^L$ and the dark exciton ±2 states equals:

$$\Delta E_{\text{AF}} = \varepsilon_{\pm1^L} - \varepsilon_{\pm2} = 2\eta + \Delta(\mu) - \sqrt{(2\eta - \Delta(\mu))^2/4 + 3\eta^2}, \tag{S5}$$

For $\Delta(\mu) < 0$ corresponding to prolate zb-CdSe NCs, the lowest dark exciton state is 0^L and ΔE_{AF} equals:

$$\Delta E_{\text{AF}} = \varepsilon_{\pm1^L} - \varepsilon_{0^L} = 2\eta - \Delta(\mu)/2 - \sqrt{(2\eta - \Delta(\mu))^2/4 + 3\eta^2}. \tag{S6}$$

S2. Dependence of the ΔE_{AF} on the NC radius

In the main manuscript, to plot ΔE_{AF} as a function of the NC radius R, we use the relationship between the exciton energy and the nanocrystal radius measured using small-angle X-ray scattering (SAXS). A general function describing the interdependence of the NC diameter and the exciton absorption peak energy at room temperature was suggested recently in Ref. [3]. We use here the simplified form of this function, extended to the liquid helium temperatures in Ref. [4]:

$$R(E) = R_1 \frac{E_0}{2\sqrt{E(E - E_0)}} \quad \text{(S7)}$$

where $E_0 = 1.826$ eV is the low-temperature optical band gap of wz-CdSe and the fitting parameter $R_1 = 2.25$ nm obtained as the fit parameter in Ref. [4].

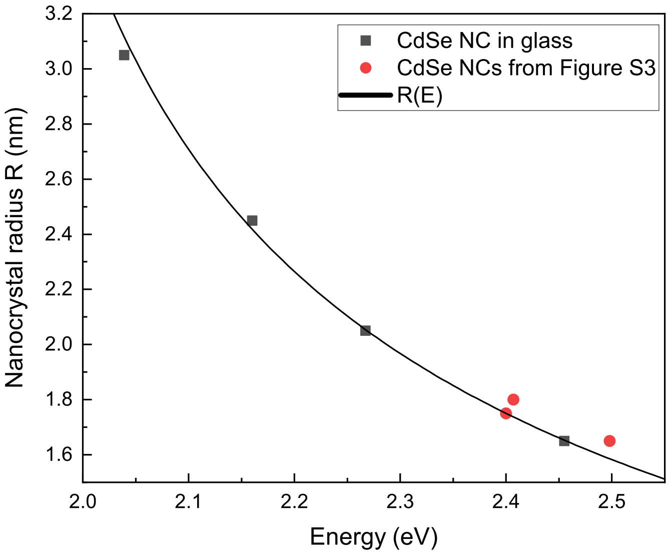

Figure S1: Fit of the low-temperature absorption data from Ref. [10] and from the current study by equation S7.

According to Ref. [5], the nanocrystal radius determined from SAXS is on average 0.25 nm larger compared to the radius determined from transmission electron microscopy. In Figure S2 we show the experimental ΔE_{AF} data from Figure 3b of the main text with R replaced by $R - 0.25$ nm. The obtained dependence agrees with Refs. [1,6]. One can see that calculated dependencies ΔE_{AF} with the standard deviation $\sigma = 0.15$ overestimate the experimental data, while $\sigma = 0.07$ fits the experimental data in the calculations for zb-CdSe NCs. For wz-CdSe NCs, the calculations with $\sigma = 0.07$ give the upper boundary for the experimental data in NCs with $R < 2$ nm.

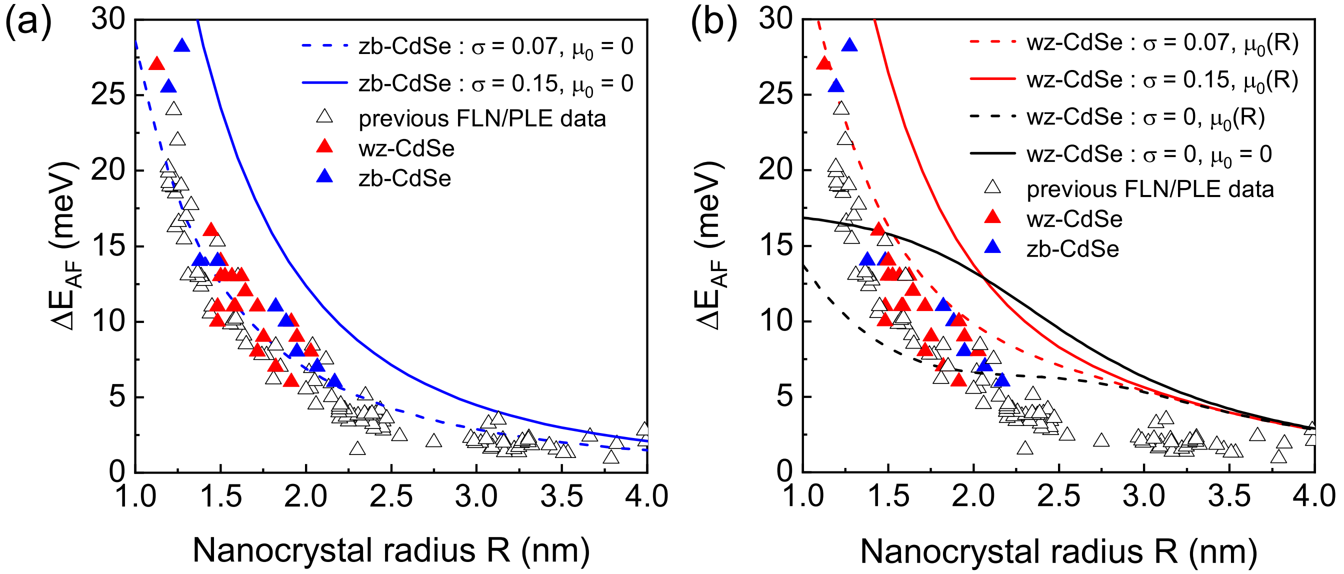

Figure S2: (a) Calculated energy of the ZPL maximum in an ensemble of zb-CdSe NCs for σ varying from 0.07 to 0.15. Symbols show the experimental FLN/PLE data from Figure 3b with the substitution $R \to R - 0.25$ nm. Here coloured triangles show the joint set of data obtained using excitation by the Hg lamp and the Ar ion laser .**(b)** Calculated energy of the ZPL maximum in an ensemble of wz-CdSe NCs with σ varying from 0.07 (red dashed line) to 0.15 (red solid line) and size dependent $\mu_0(R)$ from Ref. [1]. Solid and dashed black lines show the calculated ΔE_{AF} with $\sigma = 0$ and $\mu_0 = 0$ or $\mu_0(R)$ from Ref. [1], respectively.

S3. Non-resonant Stokes shift in CdSe and CdTe NCs

Here we present the comparison of the non-resonant Stokes shift (difference between the energies of the first maxima in the absorption and photoluminescence spectra) in zb-CdSe with $R = 1.65; 1.8$ nm and wz-CdSe NCs with $R = 1.75$ nm. As one can see in Figure S1, in all three samples a non-resonant Stokes shift of about 100 meV is observed. According to Refs. [2,6–8], the non-resonant Stokes shift in wz-CdSe NCs is equivalent to the energy difference between the initially excited bright states 0^U and $\pm 1^U$ and the emitting dark exciton state ± 2. The non-resonant Stokes shift in the zb- and wz-CdSe NCs under investigation and the previously studied wz-CdSe NCs are similar (see Figure S4). On this basis we conclude that in zb-CdSe NCs the energy fine structure of the band edge exciton should be the same as in wz-CdSe NCs. This requires an oblate shape of the zb-CdSe NCs. As one can see in Figure S4, a similar dependence of the non-resonant Stokes shift on the NC radius is observed for the zb-CdTe NCs [9].

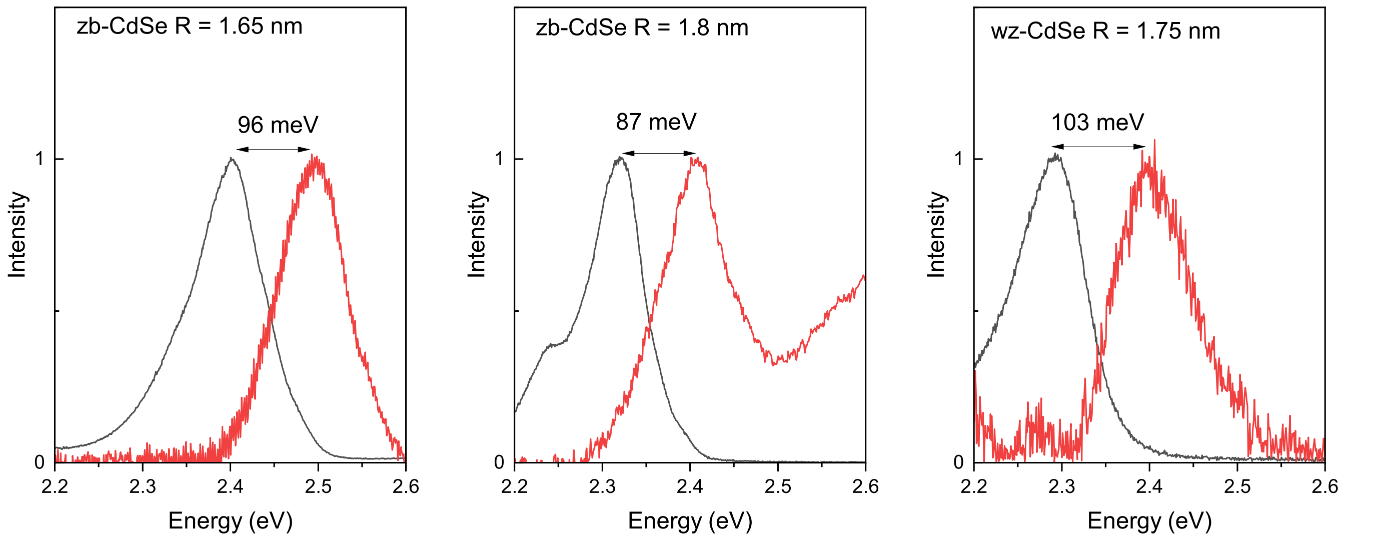

Figure S3: Low-temperature ($T = 5$ K) photoluminescence (black lines) and absorption spectra (red lines) in zb- and wz-CdSe NCs. The values of the non-resonant Stokes shifts are shown. The values of the non-resonant Stokes shifts are shown. Two PL peaks observed in zb-CdSe sample with R=1.8 nm come from a bi-modal size distribution of NCs.

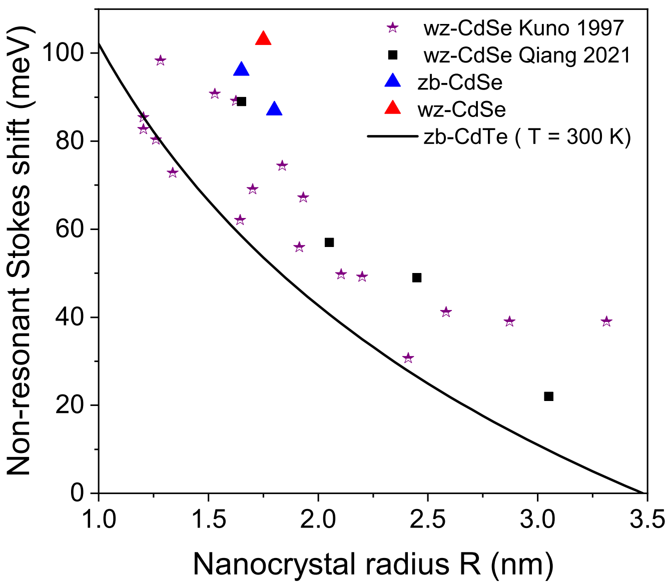

Figure S4: Size dependence of the low-temperature non-resonant Stokes shifts in wz-CdSe NCs from Refs.[6,10] and from the current study. Solid line shows the room temperature non-resonant Stokes shift in zb-CdTe NCs Ref. [9].

S4. Comparison of the bright-dark exciton splitting in CdSe and CdTe NCs

In Figure S5, we present a comparison of the data set of the $\Delta E_{\rm AF}$ from the FLN/PLE studies for CdSe NCs from Figure 3 of the main text with the data for CdTe NCs [9,11]. In Figure S5a, the $\Delta E_{\rm AF}$ is shown as function of the excitation energy by the empty triangles for the CdSe NCs and by the red circles[11] and the black squares [9] for the CdTe NCs. One simply finds that the difference between the dependencies $\Delta E_{\rm AF}$ in the CdSe and CdTe NCs is governed by the difference between the band gaps of CdSe ($E_g = 1.841$ eV) and CdTe ($E_g = 1.602$ eV). In Figure S5b, the $\Delta E_{\rm AF}$ are shown as function of the effective quantization energy $E - E_g$, resulting in the same dependence for CdSe and CdTe NCs. In Figure S5c we show the dependence $\Delta E_{\rm AF}(R)$ from Figure 3b of the paper and the dependencies for CdTe from Refs. [9,11]. Thus, one can see that for zb-CdSe NCs, wz-CdSe NCs and zb-CdTe NCs, similar $\Delta E_{\rm AF}(R)$ dependencies are observed.

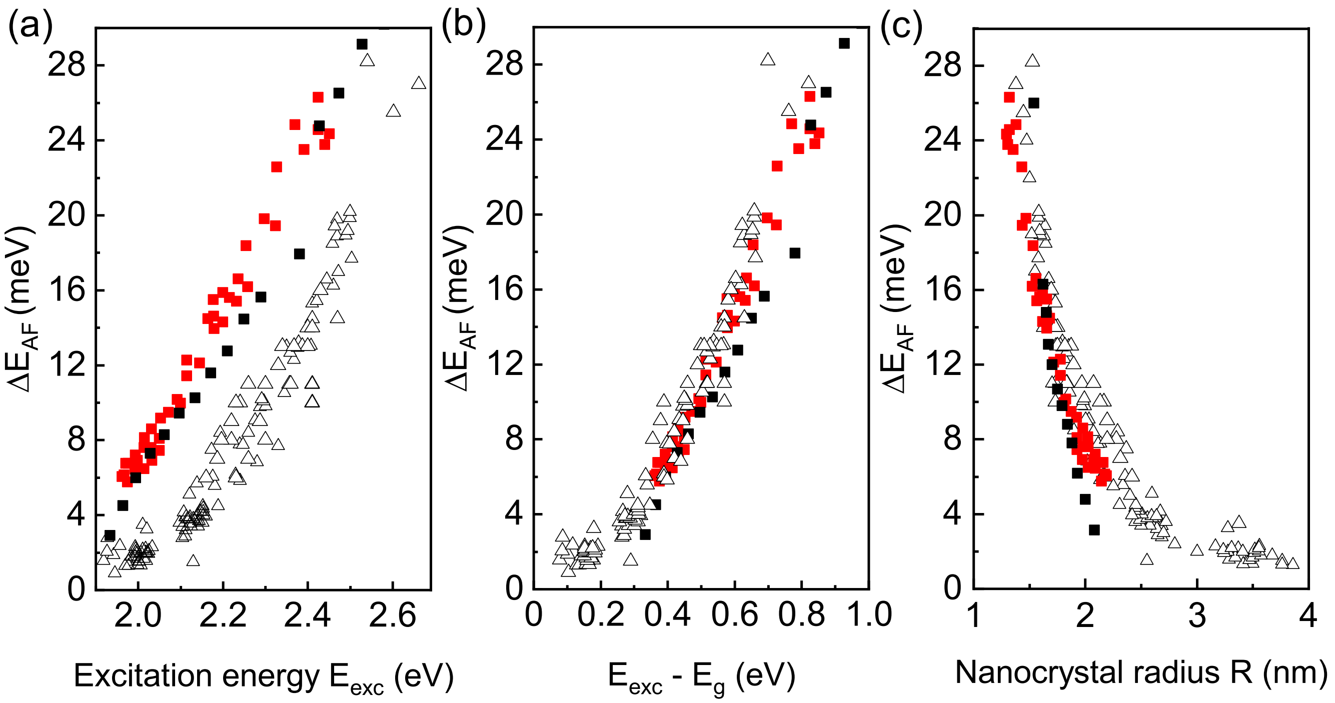

Figure S5: Dependence of the bright-dark splitting $\Delta E_{\rm AF}$ om (a) the excitation energy $E_{\rm exc}$ and (b) the effective quantization energy $E_{\rm exc} - E_g$ for CdSe NCs with $E_g = 1.841$ eV (empty triangles) and CdTe NCs with $E_g = 1.602$ eV (red [11] and black [9] squares), (c) Dependence of the $\Delta E_{\rm AF}$ on the radius of CdSe NCs and CdTe NCs.

S5.Temperature dependence of PL decay

In this section we present a comparison of fitting the temperature dependence of the long PL decay component using the three-level model [12] and the model considering acoustic phonon-induced admixture of the 0^U state to the ± 2 state [13]. In case of the three-level model, the temperature dependence of the lifetime of the long PL decay component is fitted by the equation:

$$\tau_L = \left[\frac{\Gamma_A + \Gamma_F}{2} - \frac{\Gamma_A - \Gamma_F}{2} \tanh\left(\frac{\Delta E_{\mathrm{AF}}}{2kT} \right) \right]^{-1}, \tag{S8}$$

where $\Gamma_A = \tau_A^{-1}$ and $\Gamma_F = \tau_F^{-1}$ are the recombination rates of the bright and dark exciton states, respectively. ΔE_{AF} is the energy splitting between the bright and dark exciton states, k is the Boltzmann constant. The fit parameters are $\tau_A, \tau_F, \Delta E_{\mathrm{AF}}$.

In case of the model considering acoustic phonon-induced admixture of the 0^U state to the ± 2 state, the temperature dependence of the lifetime of the long-lived PL decay component is fitted by the equation:

$$\tau_L = \tau_0 \frac{2(4\eta + \Delta)^2}{V_{ph}^2} \tanh\left(\frac{E_{\mathrm{AC}}}{2kT} \right) = \tau_{\mathrm{AC}} \tanh\left(\frac{E_{\mathrm{AC}}}{2kT} \right), \tag{S9}$$

where τ_0 is the lifetime of the 0^U state, $4\eta + \Delta$ is the energy splitting between the ± 2 and 0^U states, V_{ph} is the matrix element determining the coupling between ± 2 and 0^U due to the interaction with the acoustic phonon mode with angular momentum $l = 2$, E_{AC} is the energy of the phonon. We do not calculate V_{ph} and treat τ_{AC} as well as E_{AC} as fitting parameters.

In Figure S6 the comparison of two fitting methods is shown for the four studied samples of CdSe NCs. The fit results obtained within the three-level model are shown by the blue lines. The fit results obtained within the model considering acoustic phonon admixture are shown by the red lines. As one can see, the three-level model gives small $\Delta E_{\mathrm{AF}} \approx 3$ meV for all four samples, while $E_{\mathrm{AC}} \approx 1$ meV is close to the energy of the lowest acoustic phonon mode with $l = 2$. In two samples, the fitting is slightly improved when we combine the models with the equation for τ_L from the three-level model and the temperature dependent $\tau_F = \tau_{\mathrm{AC}} \tanh(E_{\mathrm{AC}}/2kT)$. The results of the fit using the combined model are shown by the green lines. Here we used the fixed ΔE_{AF} from the FLN/PLE studies and $\tau_A, \tau_{\mathrm{AC}}, E_{\mathrm{AC}}$ as fit parameters. One can see that the effect of the thermal population of the bright exciton states is important at $T > 20$ K. At lower temperatures, the acoustic phonon mechanism plays the major role.

Alternatively, we perform a fit of the rate of the long PL decay component, τ_L^{-1}, using two fit models. The difference between fitting τ_L and τ_L^{-1} is connected to the algorithm of the least square method. In the case of fitting τ_L, the major contribution to the fit error occurs at low temperatures where τ_L is long. In the case of fitting τ_L^{-1}, the major contribution to the fit error comes at high temperatures where the rate τ_L^{-1} is fast.

As one can see in Figure S7, fitting τ_L^{-1} within the three-level model results in an almost twice larger ΔE_{AF}, as compared to the ΔE_{AF} obtained from fitting τ_L. With the acoustic phonon model, only in the zb-CdSe sample with R=1.65 nm we obtain the parameters E_{AC} and τ_{AC} with reasonable accuracy. In the other samples the error for these parameters is of the order or exceeds E_{AC} and τ_{AC}, in contrast to fitting τ_L. For this reason, we used the τ_{AC} determined from fitting of τ_L, and E_{AC} was used as the only fit parameter.

We additionally compared these two fit models using the τ_L data for the zb- and wz-CdSe NCs from Ref. [10,12,14] (see Figure S8-S11). Again, one can see that the ΔE_{AF} from fitting τ_L^{-1} are almost twice larger compared to the values from fitting of τ_L.

The dependence of the energy E_{AC} determined from the acoustic phonon model [13] on the

CdSe NC radius is shown by the symbols in Figure S12. The black line in Figure S12 shows the NC size dependence of the energy of the acoustic phonon mode $n = 0, l = 2$, calculated within the Lamb theory for spherical NCs with free boundary conditions. In the whole range on NC sizes, the E_{AC} values are 0.5 meV below the theoretical estimation. Probably there are some additional temperature dependent activation mechanism of the dark exciton recombination.

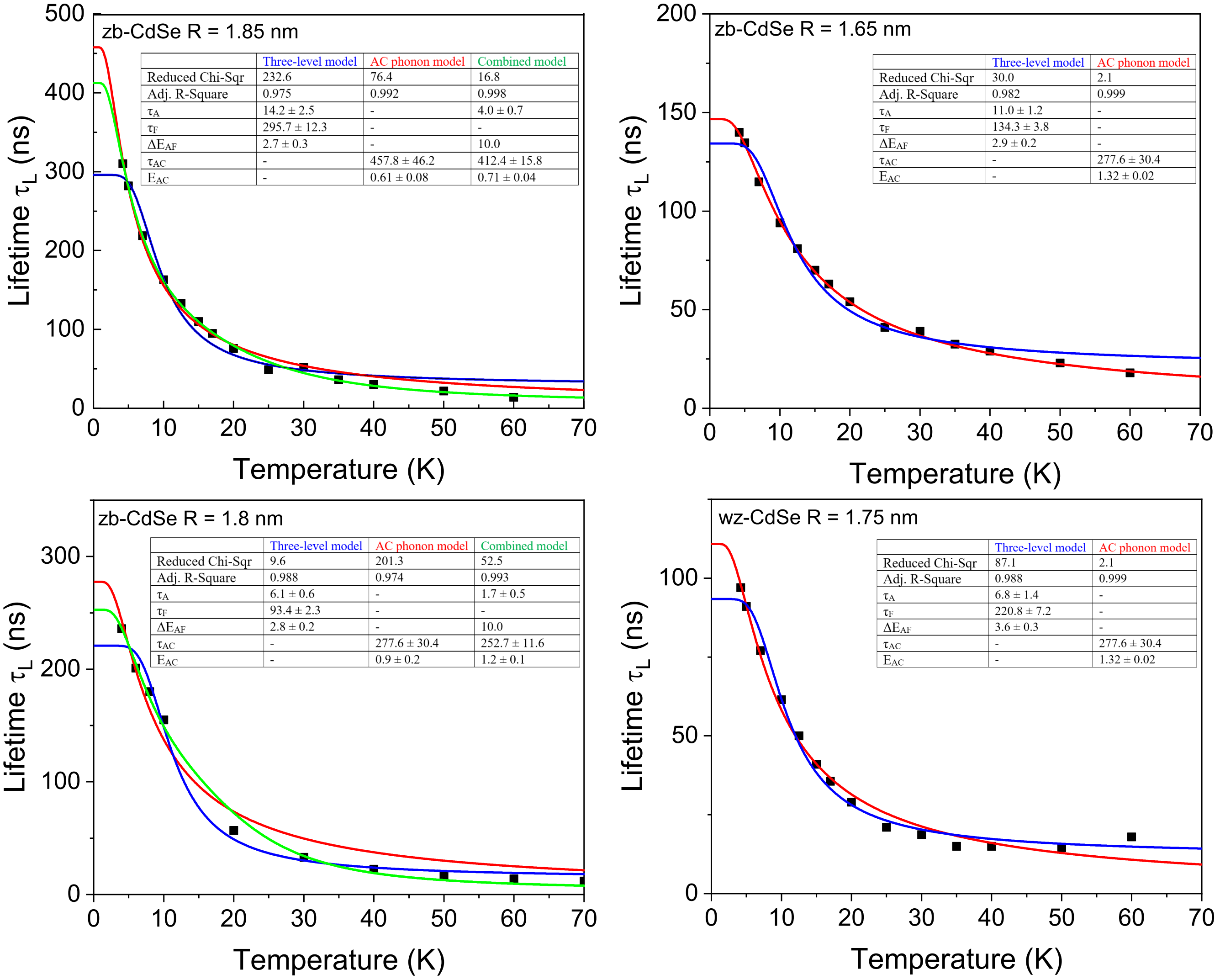

Figure S6: Experimental temperature dependence of the long PL decay time τ_L (symbols) in zb-CdSe and wz-CdSe NCs. Blue lines are obtained by fitting of the experimental data points by equation S8 within the three level model [12]. Red lines are obtained by performing the fit by equation S9 within the model considering acoustic phonon-induced activation of the dark exciton emission [13]. Green lines show the result of the fit by equation S8 with τ_F described by equation S9 and fixed ΔE_{AF} as determined from FLN/PLE decay studies.

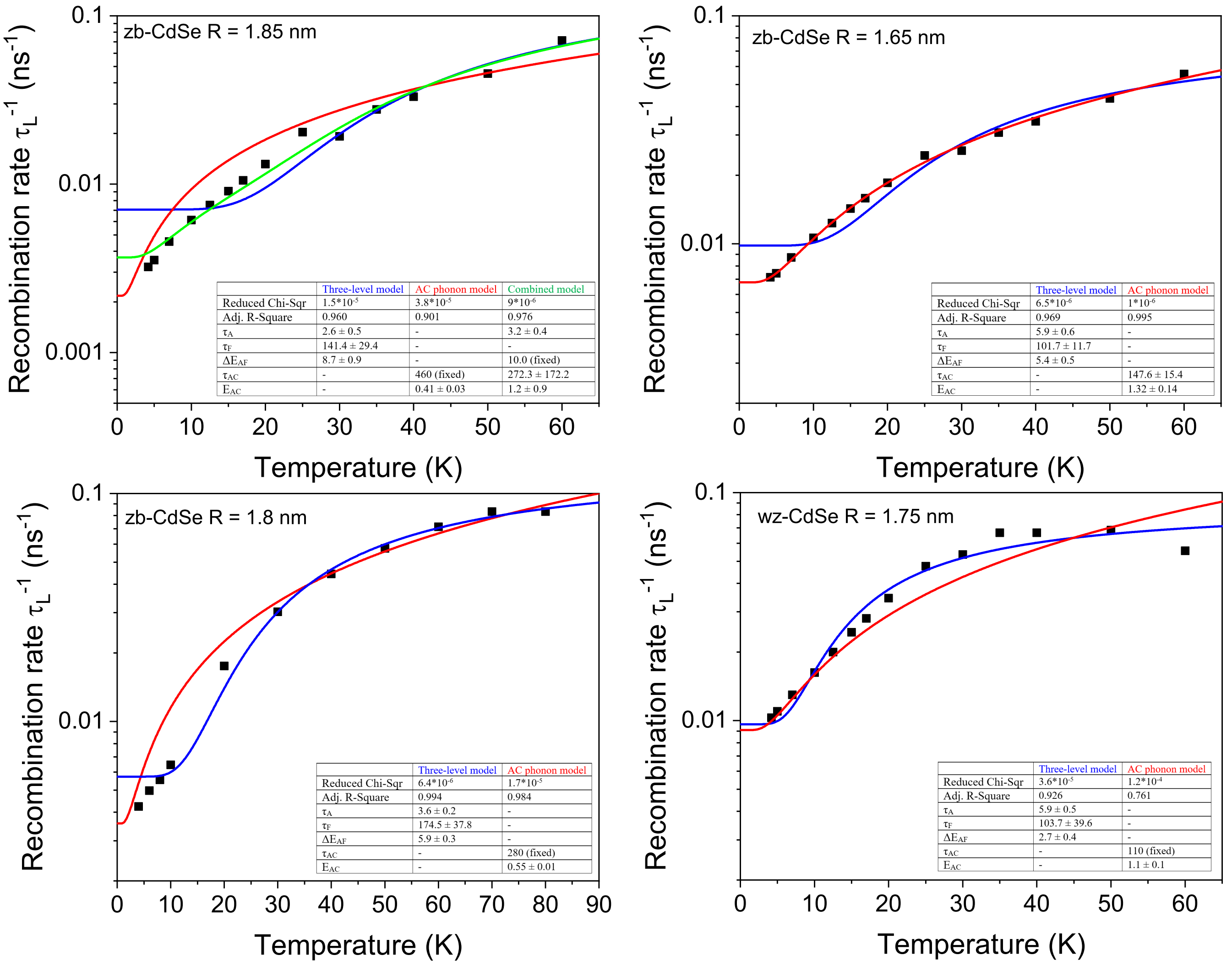

zb-CdSe R = 1.85 nm

	Three-level model	AC phonon model	Combined model
Reduced Chi-Sqr	$1.5*10^{-5}$	$3.8*10^{-5}$	$9*10^{-6}$
Adj. R-Square	0.960	0.901	0.976
τ_A	2.6 ± 0.5	-	3.2 ± 0.4
τ_F	141.4 ± 29.4	-	-
ΔE_{AF}	8.7 ± 0.9	-	10.0 (fixed)
τ_{AC}	-	460 (fixed)	272.3 ± 172.2
E_{AC}	-	0.41 ± 0.03	1.2 ± 0.9

zb-CdSe R = 1.65 nm

	Three-level model	AC phonon model
Reduced Chi-Sqr	$6.5*10^{-6}$	$1*10^{-6}$
Adj. R-Square	0.969	0.995
τ_A	5.9 ± 0.6	-
τ_F	101.7 ± 11.7	-
ΔE_{AF}	5.4 ± 0.5	-
τ_{AC}	-	147.6 ± 15.4
E_{AC}	-	1.32 ± 0.14

zb-CdSe R = 1.8 nm

	Three-level model	AC phonon model
Reduced Chi-Sqr	$6.4*10^{-6}$	$1.7*10^{-5}$
Adj. R-Square	0.994	0.984
τ_A	3.6 ± 0.2	-
τ_F	174.5 ± 37.8	-
ΔE_{AF}	5.9 ± 0.3	-
τ_{AC}	-	280 (fixed)
E_{AC}	-	0.55 ± 0.01

wz-CdSe R = 1.75 nm

	Three-level model	AC phonon model
Reduced Chi-Sqr	$3.6*10^{-5}$	$1.2*10^{-4}$
Adj. R-Square	0.926	0.761
τ_A	5.9 ± 0.5	-
τ_F	103.7 ± 39.6	-
ΔE_{AF}	2.7 ± 0.4	-
τ_{AC}	-	110 (fixed)
E_{AC}	-	1.1 ± 0.1

Figure S7: Experimental temperature dependence of the long PL decay rate τ_L^{-1} (symbols) in zb-CdSe and wz-CdSe NCs. Blue lines are obtained by fitting of the experimental data points by equation S8 within the three level model [12]. Red lines are obtained by performing the fit by equation S9 within the model considering acoustic phonon-induced activation of the dark exciton emission [13]. Green lines show the result of the fit by equation S8 with τ_F described by equation S9 and fixed ΔE_{AF} as determined from FLN/PLE decay studies.

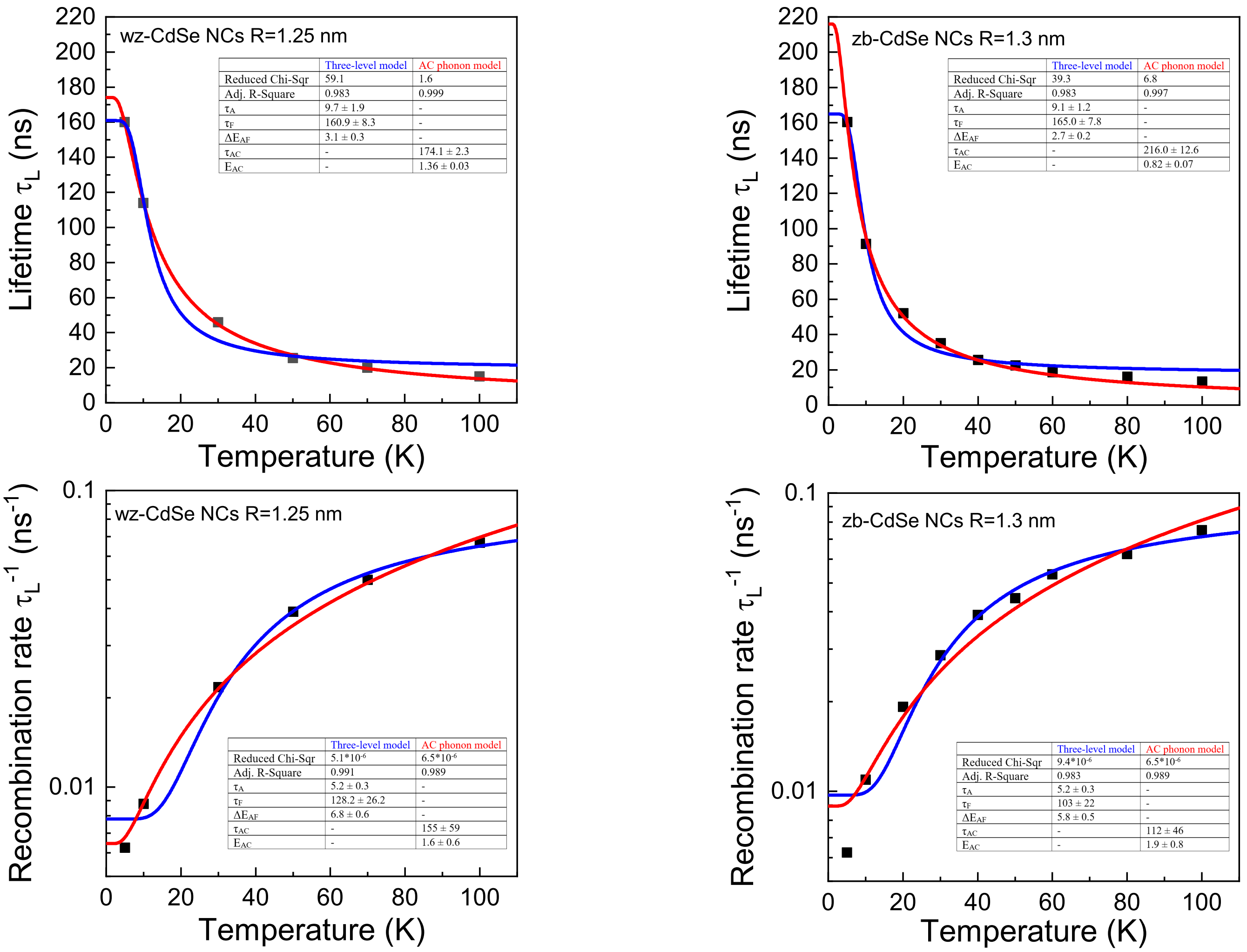

Figure S8: Fits to the τ_L and τ_L^{-1} data from Ref. [14], using the three-level model (blue lines) and the acoustic phonon-induced activation model (red lines) from Ref. [13].

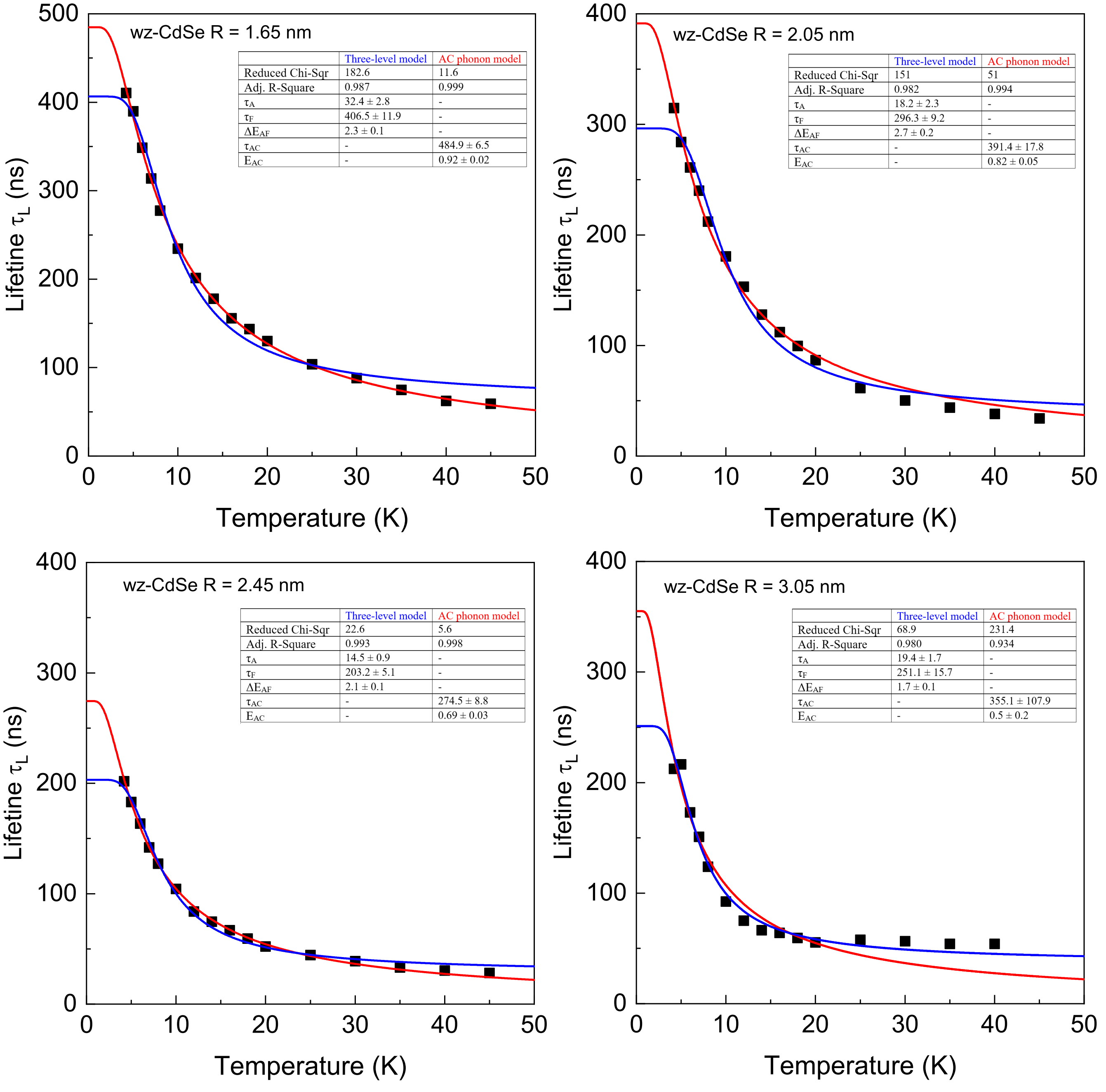

Figure S9: Fits to the τ_L data in wz-CdSe NCs with $R = 1.65 - 3.05$ nm from Ref. [10], using the three-level model (blue lines) and the acoustic phonon-induced activation model (red lines) from Ref. [13].

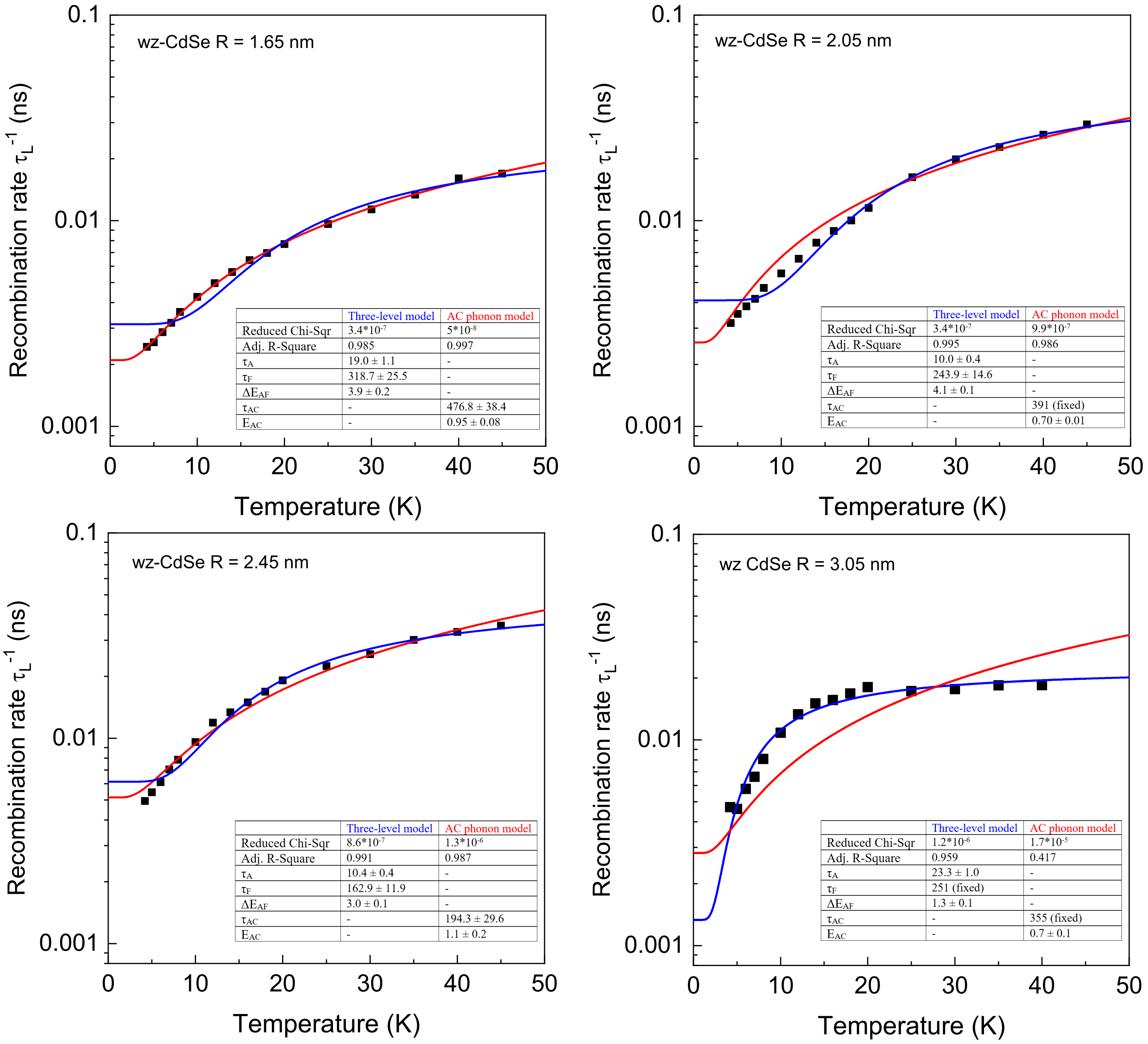

Figure S10: Fit to the τ_L^{-1} data in wz-CdSe NCs with $R = 1.65 - 3.05$ nm from Ref. [10], using the three-level model (blue lines) and the acoustic phonon-induced activation model (red lines) from Ref. [13].

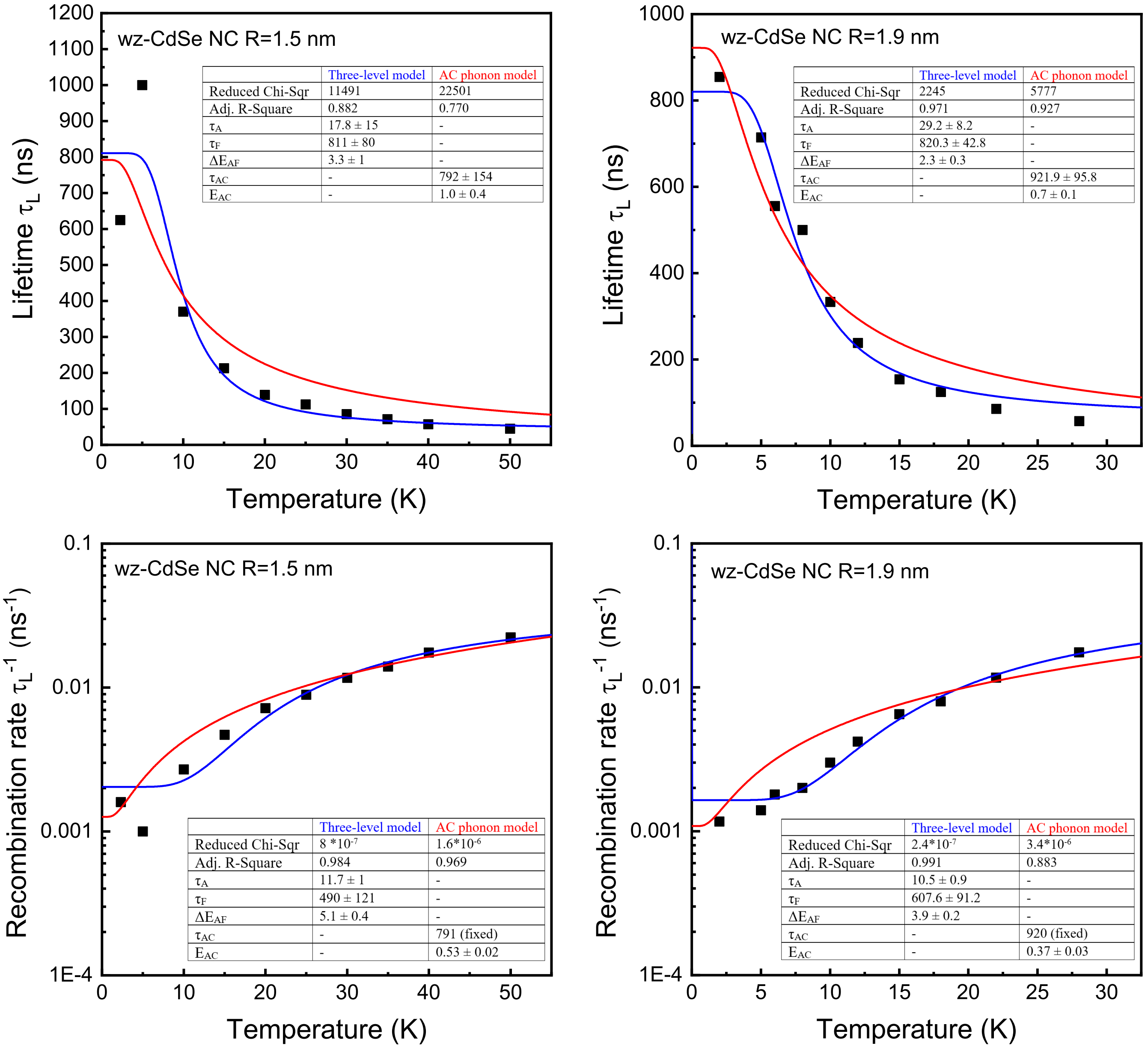

Figure S11: Fits to the τ_L and τ_L^{-1} data measured on single CdSe NCs in Ref. [12], using the three-level model (blue lines) and the acoustic phonon-induced activation model (red lines) from Ref. [13]

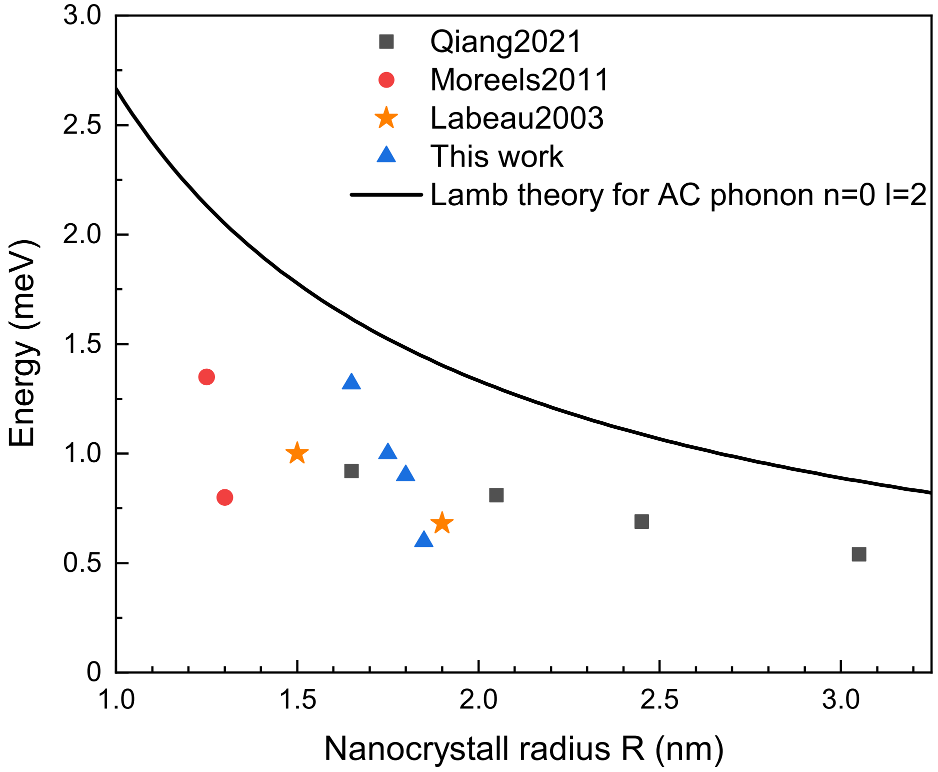

Figure S12: Dependence of the energy E_{AC} determined from fitting the $\tau_L(T)$ dependence within the model of acoustic phonon-induced activation. Solid line corresponds to the energy of the acoustic phonon mode with $n = 0, l = 2$ in a CdSe spherical nanocrystal, calculated using Lamb's theory with free boundary conditions. Red circles [14], black squares [10], and orange stars [12] show the E_{AC} values obtained by fitting the $\tau_L(T)$ data from previous studies within the acoustic phonon activation model. Blue triangles correspond to E_{AC} values determined in this study (see red lines in Figure S6).

References

1. Efros, Al. L.; Rosen, M.; Kuno, M.; Nirmal, M.; Norris, D. J.; Bawendi, M. Band-edge exciton in quantum dots of semiconductors with a degenerate valence band: Dark and bright exciton states. *Phys. Rev. B* **1996**, *54*, 4843.

2. Sercel, P. C.; Efros, A. L. Band-edge exciton in CdSe and other II–VI and III–V compound semiconductor nanocrystals - revisited. *Nano Lett.* **2018**, *18*, 4061–4068.

3. Aubert, T.; Golovatenko, A. A.; Samoli, M.; Lermusiaux, L.; Zinn, T.; Ab´ecassis, B.; Rodina, A. V.; Hens, Z. General expression for the size-dependent optical properties of quantum dots. *Nano Lett.* **2022**, *22*, 1778–1785.

4. Qiang, G.; Zhukov, E.; Evers, E.; Yakovlev, D.; Golovatenko, A.; Rodina, A.; Onushchenko, A.; Bayer, M. Electron spin coherence in CdSe nanocrystals in a glass matrix. *ACS Nano* **2022**, *16* 18838–18848.

5. Kuno, M.K. Band edge spectroscopy of CdSe quantum dots. Doctoral dissertation, Massachusetts Institute of Technology, Cambridge, 1998.

6. Kuno, M.; Lee, J.-K.; Dabbousi, B. O.; Mikulec, F. V.; Bawendi, M. G. The band edge luminescence of surface modified CdSe nanocrystallites: Probing the luminescing state. *J. Chem. Phys* **1997**, 106, 9869.

7. Gupalov, S. V.; Ivchenko, E. L. The fine structure of excitonic levels in CdSe nanocrystals. *Phys. Solid State* **2000**, *42*, 2030–2038.

8. Norris, D.; Efros, Al. L.; Rosen, M.; Bawendi, M. Size dependence of exciton fine structure in CdSe quantum dots. *Phys. Rev. B* **1996**, 53, 16347.

9. Zhong, H.; Nagy, M.; Jones, M.; Scholes, G.D. Electronic states and exciton fine structure in colloidal CdTe nanocrystals. *J. Phys. Chem. C* **2009**, 113, 10465–10470.

10. Qiang, G.; Golovatenko, A. A.; Shornikova, E. V.; Yakovlev, D. R.; Rodina, A. V.; Zhukov, E. A.; Kalitukha, I. V.; Sapega, V. F.; Kaibyshev, V. K.; Prosnikov, M. A.; Christianen, P. C. M.; Onushchenko, A. A.; Bayer, M. Polarized emission of CdSe nanocrystals in magnetic field: the role of phonon-assisted recombination of the dark exciton. *Nanoscale* **2021**, 13, 790–800.

11. Lavallard, P.; Chamarro, M.; Pérez-Conde, J.; Bhattacharjee, A. K.; Goupalov, S. V.; Lipovskii, A. A. Exchange interaction and acoustical phonon modes in CdTe nanocrystals. *Solid State Comm.* **2003**, 127, 439–442.

12. Labeau, O.; Tamarat, P.; Lounis, B. Temperature dependence of the luminescence lifetime of single CdSe/ZnS quantum dots. *Phys. Rev. Lett.* **2003**, 90, 257404.

13. Rodina, A. V.; Efros, Al. L. Radiative recombination from dark excitons in nanocrystals: Activation mechanisms and polarization properties. *Phys. Rev. B* **2016**, 93, 155427.

14. Moreels, I.; Rainó, G.; Gomes, R.; Hens, Z.; Stöferle, T.; Mahrt, R. F. Band-edge exciton fine structure of small, nearly spherical colloidal CdSe/ZnS quantum dots. *ACS Nano* **2011**, 5, 8033–8039.
